# The AAA + ATPase valosin-containing protein (VCP)/p97/Cdc48 interaction network in *Leishmania*

**DOI:** 10.1038/s41598-020-70010-4

**Published:** 2020-08-04

**Authors:** Bruno Guedes Aguiar, Carole Dumas, Halim Maaroufi, Prasad K. Padmanabhan, Barbara Papadopoulou

**Affiliations:** 1 Division of Infectious Disease and Immunity, CHU de Quebec Research Center-Laval University, 2705 Laurier Blvd, Quebec, QC G1V 4G2 Canada; 2 Department of Microbiology-Infectious Disease and Immunology, Faculty of Medicine, University Laval, Quebec, QC G1V 4G2 Canada; 3 Present Address: Department of Community Medicine, Federal University of Piauí, Teresina, Brazil; 4 Institut de Biologie Intégrative Et Des Systèmes (IBIS), Laval University, Quebec, QC Canada

**Keywords:** Microbiology, Parasite biology, Proteomics, Protein-protein interaction networks, Gene ontology, Network topology, Protein structure predictions

## Abstract

Valosin‐containing protein (VCP)/p97/Cdc48 is an AAA + ATPase associated with many ubiquitin-dependent cellular pathways that are central to protein quality control. VCP binds various cofactors, which determine pathway selectivity and substrate processing. Here, we used co-immunoprecipitation and mass spectrometry studies coupled to in silico analyses to identify the *Leishmania infantum* VCP (*Li*VCP) interactome and to predict molecular interactions between *Li*VCP and its major cofactors. Our data support a largely conserved VCP protein network in *Leishmania* including known but also novel interaction partners. Network proteomics analysis confirmed *Li*VCP-cofactor interactions and provided novel insights into cofactor-specific partners and the diversity of *Li*VCP complexes, including the well-characterized VCP*-*UFD1-NPL4 complex. Gene Ontology analysis coupled with digitonin fractionation and immunofluorescence studies support cofactor subcellular compartmentalization with either cytoplasmic or organellar or vacuolar localization. Furthermore, in silico models based on 3D homology modeling and protein–protein docking indicated that the conserved binding modules of *Li*VCP cofactors, except for NPL4, interact with specific binding sites in the hexameric *Li*VCP protein, similarly to their eukaryotic orthologs. Altogether, these results allowed us to build the first VCP protein interaction network in parasitic protozoa through the identification of known and novel interacting partners potentially associated with distinct VCP complexes.

## Introduction

*Leishmania* species cause a large spectrum of diseases in humans ranging from skin lesions to visceral damage, which is lethal, if left untreated. Treatment options for leishmaniasis are limited and toxic and no effective vaccine is currently available (https://www.dndi.org/ diseases-projects/leishmaniasis/). Within its mammalian host, *Leishmania* replicates in the phagolysosome compartment of macrophages where it encounters various stress stimuli that trigger important changes in gene expression^1–5^ and parasite metabolism^6,7^. Most of those stresses lead to DNA damage or protein misfolding that has to be corrected. As many other eukaryotes^8^, *Leishmania* has evolved quality control systems that cooperate to eliminate damaged proteins^9–12^.

Recently, we have undertaken studies to characterize the *Leishmania* valosin-containing protein (VCP)/p97/Cdc48 ortholog (VCP and p97 in metazoa, Cdc48 in yeast)^10^, one of the key quality control components in recycling or degrading misfolded proteins or aggregates. VCP belongs to the AAA + (Associated with diverse cellular Activities) family of ATPases that hydrolyze ATP and use the resulting energy to extract polyubiquitinated target proteins from membranes, organelles, and large protein assemblies and delivered them to proteasomal degradation^13–16^. As a central component of the Ubiquitin Proteasome System (UPS), VCP/p97 plays a critical role in cellular proteostasis^13,17,18^. Indeed, VCP/p97 is involved in the endoplasmic reticulum (ER)-associated protein degradation^15,19^, the mitochondrion-associated protein degradation^20^, ribosomal quality control^21^, the extraction of chromatin-bound proteins^22^ or of damaged lysosomes by autophagy^23^, genome stability^24^, and stress granules clearance^25^. Mutations in this well-conserved protein can lead to protein aggregation and have been linked to several diseases, including neurodegenerative and muscular disorders and cancer^26–28^.

Each monomer of the hexameric VCP/p97 protein is composed by an N-terminal domain followed by two tandem ATPase domains (D1 and D2) separated by a short linker, and an unstructured C-terminal tail^29^. The N-terminal domain can be further subdivided into two subdomains, Nn (15–95 aa) and Nc (104–175 aa). This structure allows the association of VCP with a large variety of cofactors/adaptors which determine substrate specificity, target the ATPase to different cellular locations, or modify the ubiquitin chain attached to the substrate^30–32^. So far, about 30–40 cofactors have been identified in mammals but their exact functions are still poorly understood. Whether they have a substrate-recruiting, processing or regulatory function, most cofactors interact with the Nn or Nc subdomains of VCP via a small number of conserved binding modules, while a lower number binds to the unstructured C‐terminal tail formed by the last 7 amino acids^30–32^. In mammals, most VCP cofactors contain the ubiquitin regulatory X domain (UBX) or UBX-like (UBXL) with similar three-dimensional structure described for ubiquitin. The UBX module interacts with the Nn-Nc cleft of the VCP through the Rx(3)FPR motif. Proteins such as UBXD1 to UBXD6, UBXD9 and UBXD11 contain only a UBX domain. The UBA (ubiquitin associated)-UBX cofactors, such as UBXD7, UBXD8 (FAF2), UBXD10 (p47), UBXD12 (FAF1) and UBXD13 also accommodate an UBA domain that is fundamental for interacting with ubiquitinated substrates^16,33^. Cofactors such as p47 harbor, in addition to the UBX and UBA domains important for their function in the UPS, a SHP (BS1, binding segment 1) motif as another site for interaction with the Nc subdomain of VCP^31,32^. Cofactors harboring either a VIM (VCP-interacting motif) or VBM (VCP-binding motif) motif also interact with the same hydrophobic pocket of the N-domain^32^. One of the most studied VCP cofactor, the heterodimer UFD1-NPL4 (UN), interacts with VCP to form the VCP-UFD1-NPL4 complex which extracts polyubiquitinated proteins from membranes and macromolecular complexes and is involved in a series of biological processes, including ER-associated degradation (ERAD)^34^. Few cofactors have been reported to interact with the C-terminus of VCP. These harbor a PUB (PNGase/UBA or UBX-containing proteins) or a PUL (PLAP, Ufd3p, and Lub1p) domain which forms a hydrophobic pocket for interactions with the C-terminal tail of VCP^35^ following association of key amino acids such as Leu^804^ and the aromatic side chain of the penultimate tyrosine^805^ residue^30–32^.

Our initial studies on the ubiquitin selective chaperone VCP/p97 in *Leishmania* (*Li*VCP) demonstrated its essential role in the parasite intracellular development and survival under heat stress^10^. In this study, we provide novel insights into the *Li*VCP interaction network. A series of immunoprecipitation experiments coupled to liquid chromatography-tandem mass spectrometry analysis were used to identify the major interacting partners of *Li*VCP. These studies uncovered p47, UFD1, NPL4, FAF2 and PUB1 as the core *Li*VCP cofactors. Network proteomics for each cofactor confirmed close partnership with *Li*VCP and revealed the presence of multiple *Li*VCP complexes in *Leishmania*, including the well-characterized *Li*VCP*-Li*UFD1-*Li*NPL4 complex. Gene Ontology analysis of each cofactor proteome combined with digitonin fractionation and immunofluorescence studies support cofactor subcellular compartmentalization. Furthermore, in silico models based on 3D homology modeling and protein–protein docking predicted that, with the exception of NPL4, conserved binding modules within key *Leishmania* VCP cofactors interact with specific binding sites in the hexameric *Li*VCP protein, as described for their mammalian orthologs. Altogether, these results allowed us to build the *Leishmania* VCP protein network, the first characterized in parasitic protozoa.

## Results

### Identification of the *Leishmania* VCP/p97 cofactors and core proteome

VCP/p97 is a hexameric protein that can interact with a large number of protein cofactors through its N-terminal and C-terminal domains^27^. These interactions are key for its activity and functional diversity. This study aimed to identify the *Leishmania* VCP interactome based on the human VCP/p97 network from the BioGrid database (Supplementary Table S1) as well as on protein–protein interaction experiments. According to the e-value similarity accessed by protein BLAST (blastP), most proteins from the human VCP network have homologs in *Leishmania* (Supplementary Fig. S1).

**Figure 1:**
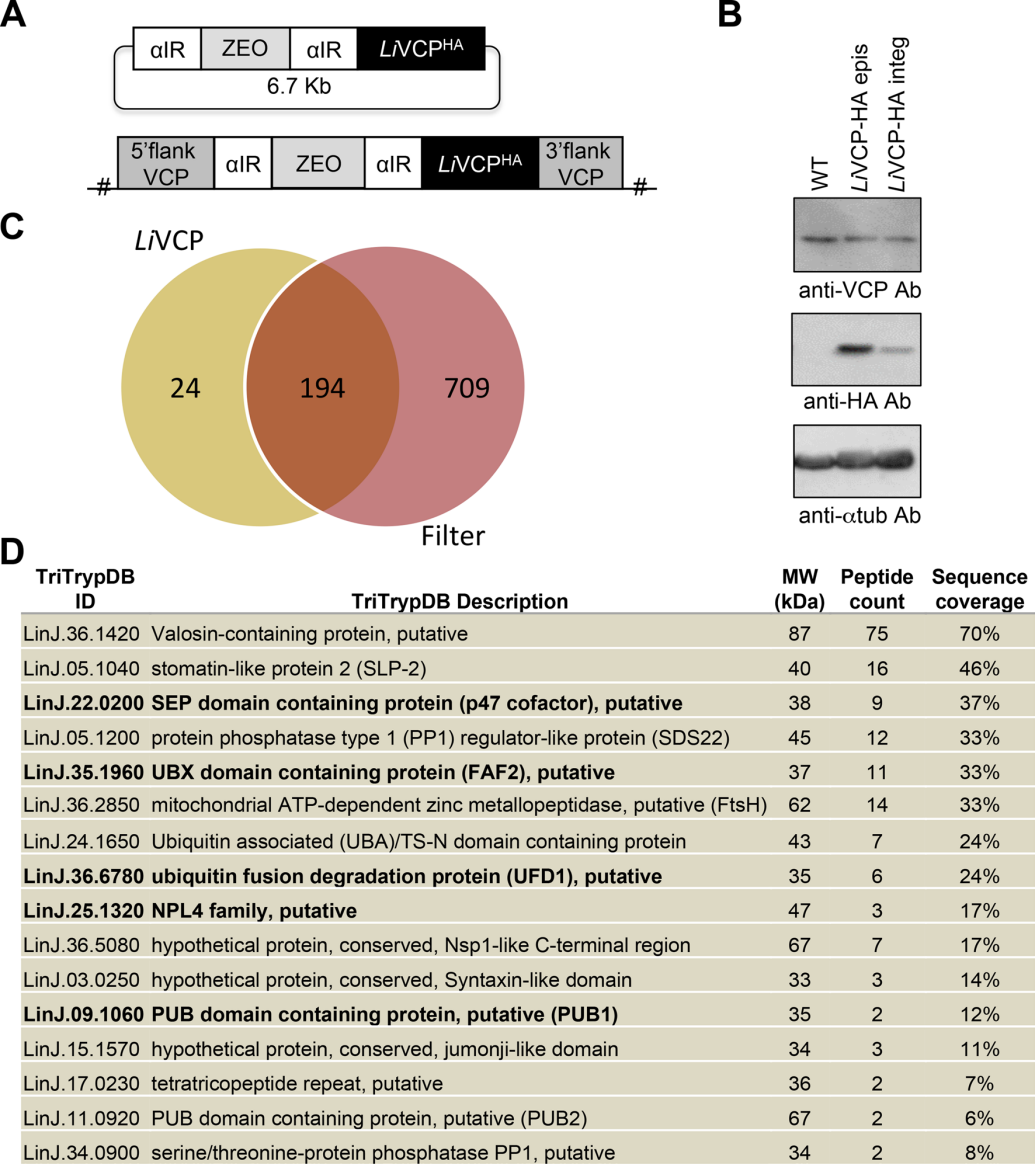
The *Leishmania Li*VCP core partners identified by co-immunoprecipitation and mass spectrometry studies. (**A**) Schematic draw of the construct used to express the *L. infantum* valosin‐containing protein (*Li*VCP) tagged with an HA epitope at the C‐terminus (VCP^HA^) either episomally or integrated into the *L. infantum* VCP endogenous locus. (**B**) Western blotting to evaluate *Li*VCP-HA expression levels using anti-*Tb*VCP and anti-HA antibodies. The alpha-tubulin antibody was used as protein loading control. The uncropped blots are shown in Supplementary Fig. S20 (panel A). (**C**) Venn diagram demonstrating the number of proteins identified by mass spectrometry in *Li*VCP-HA immunoprecipitates of seven independent experiments that were filtered against 5 unrelated HA-tagged proteins (for more details see Methods and Supplementary Table S2). (**D**) *Li*VCP core partners identified in (C) with an average of exclusive unique peptide count (EUPC) of ≥ 2. The five *Li*VCP core cofactors studied here (*Li*p47, *Li*UFD1, *Li*NPL4, *Li*FAF2 and *Li*PUB1) are indicated in bold. For the complete list, see Supplementary Table S2.

Seeking for the identification of *Leishmania* VCP cofactors, we carried out a series of immunoprecipitation (IP) experiments with either C- or N-terminally HA-tagged *Leishmania infantum* VCP (*Li*VCP; LinJ.36.1420)^10^ followed by LC–MS/MS, database searching, and peptide identification. To allow VCP expression from its endogenous locus, we replaced one *Li*VCP genomic copy by a HA-epitope tagged *Li*VCP (*Li*VCP-HA) (Fig. 1A). Both episomal and integrated *Li*VCP-HA versions were expressed at levels comparable to the endogenous VCP protein (Fig. 1B) and yielded similar IP results. From seven independent IP-MS/MS experiments (see Methods), 218 proteins were initially identified as potential *Li*VCP interacting partners (Supplementary Table S2). To decrease the number of false positives in the *Li*VCP network building, we applied a stringent filter. First, we calculated the average number of peptides for each protein in all seven IPs and then we excluded proteins also found in five independent IP-MS/MS studies conducted with HA-tagged proteins unrelated to the VCP complex (Fig. 1C and Supplementary Table S2). From this analysis, 24 proteins were selected to specifically associate with *Li*VCP. Among those, 15 proteins with an average of exclusive unique peptide count (EUPC) ≥ 2.0 were classified as the *Li*VCP core partners (Fig. 1D and Supplementary Table S2). These share conserved domains with known VCP cofactors in other eukaryotes, such as the p47 (Shp1) UBX/UBA- and SEP-containing protein (LinJ.22.0200), the UBX-containing protein FAF (Fas-associated factor) shown a higher homology to the human FAF2 protein (UBXD8 or ETEA; LinJ.35.1960), the ubiquitin fusion degradation protein 1 (UFD1; LinJ.36.6780), the nuclear protein localization 4 (NLP4; LinJ.25.1320) known to form a heterodimer with UFD1^34^, an ubiquitin associated UBA/TS-N domain protein (LinJ.24.1650) with an identified ER membrane signal peptide, and two PUB (PNGase/UBA or UBX) domain containing proteins (LinJ.11.0920 and LinJ.09.1060). In addition, the *Li*VCP proteome core includes the serine/threonine phosphatase type 1 (PP1) (LinJ.34.0900), the PP1 regulator-like protein (LinJ.05.1200) whose human homolog SDS22 is known to interact with VCP^36^, three mitochondrial proteins—a stomatin-like protein (LinJ.05.1040), an ATP-dependent zinc metallopeptidase (LinJ.36.2850) and the AAA + FtsH protease (LinJ.36.2850), a tetratricopeptide repeat protein part of the ER membrane protein complex subunit 2 (LinJ.17.0230) and three hypothetical proteins (LinJ.36.5080, LinJ.03.0250, LinJ.15.1570) (Fig. 1D) with some similarity to the yeast nuclear envelope protein Nsp1-like C-terminal region, syntaxin-like domain, and putative nuclear jumonji-like domain, respectively.

Apart from the above core partners, *Li*VCP co-immunoprecipitated heat shock proteins, chaperonins, T-complex proteins, mitochondrial integrity and stress response proteins, translation factors and ribosomal proteins, components of the trypanothione and peroxidase systems, RNA-binding proteins, and proteasome subunits (Supplementary Table S2) in line with its function in a broad array of ubiquitin-dependent protein quality control pathways^13–16^.

### ‘Network proteomics’ analysis to identify interacting partners of the core *Li*VCP cofactors and associations between cofactor-bound proteins

**Figure 2:**
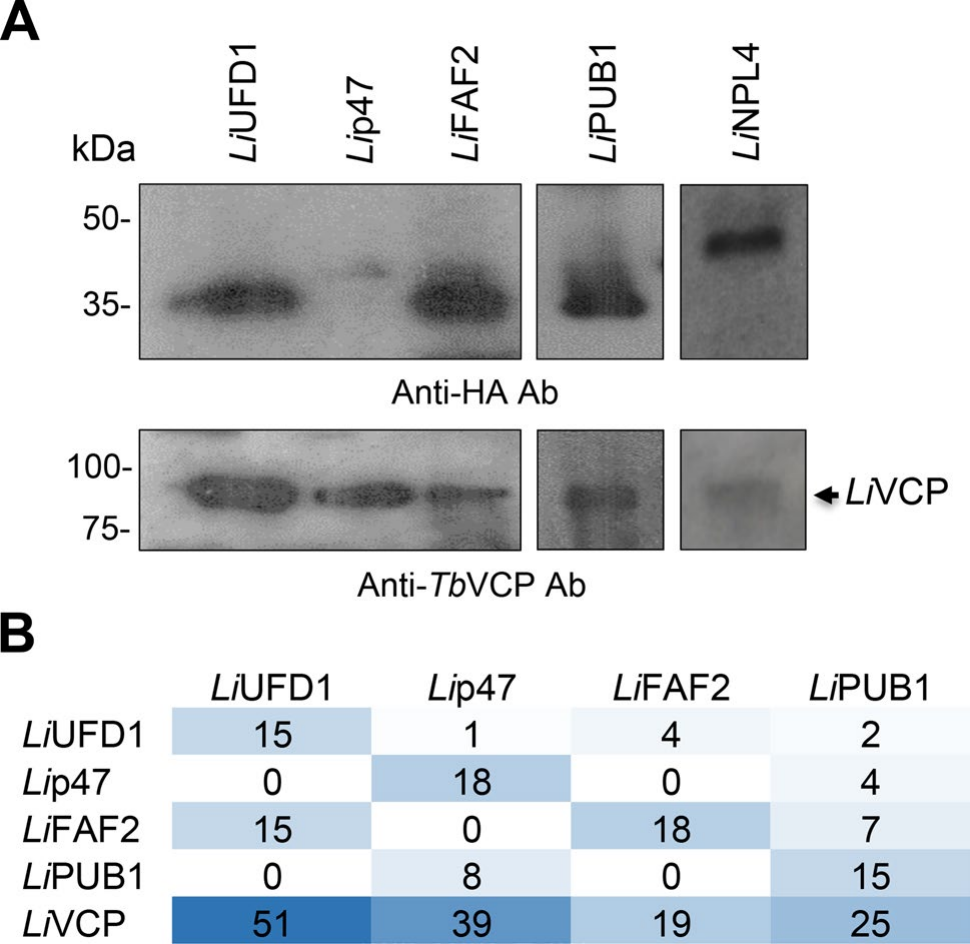
Reciprocal interactions between the *Leishmania Li*VCP and its key cofactors. (**A**) *Li*VCP detection by western blotting with an anti-*Tb*VCP antibody following immunoprecipitation of C- or N- terminally HA-tagged *Li*UFD1, *Li*p47, *Li*FAF2, *Li*NPL4 and *Li*PUB1 proteins ectopically expressed in *Leishmania*. The uncropped blots derived from different gels are shown in Supplementary Fig. S20 (panels B-D) together with other repetitions of this experiment. (**B**) Detection of *Li*VCP and its cofactors *Li*UFD1, *Li*p47, *Li*FAF2 and *Li*PUB1 by mass spectrometry after co-immunoprecipitation using anti-HA magnetic beads for recombinant *Leishmania* expressing C- or N- terminally HA-tagged cofactor proteins. The average of EUPC for two independent experiments for each cofactor is shown here. For the complete list of mass spectrometry results, see Supplementary Table S2.

The interaction of p47, UFD1, NPL4, FAF2 and PUB1 with the *Leishmania* VCP ortholog was further confirmed by inverse co-immunoprecipitation studies. C- or N- terminally HA-tagged *Li*p47, *Li*NPL4, *Li*UFD1, *Li*FAF2, or *Li*PUB1 proteins cloned into vector pSP $\alpha$ ZEO $\alpha$ were transfected and stably expressed in *L. infantum* promastigotes (Supplementary Fig. S2A). Immunoprecipitation of these HA-tagged cofactor versions using an anti-HA antibody followed by western blotting with a specific anti-*Tb*VCP antibody^10,37^ confirmed their association with *Li*VCP (Fig. 2A). The co-immunoprecipitation of *Li*UFD1, *Li*NPL4, *Li*p47, *Li*FAF2 and *Li*PUB1 with *Li*VCP (Fig. 1D and Table 1) and the detection of *Li*VCP in *Li*UFD1, *Li*NPL4, *Li*p47, *Li*FAF2 and *Li*PUB1 immunoprecipitates by western blotting (Fig. 2A) and mass spectrometry (Fig. 2B, Table 1, Supplementary Table S2) confirmed their close partnership.

**Table 1:** Proteins specifically associated with one or more *Leishmania Li*VCP cofactors as determined by immunoprecipitation and LC–MS/MS studies.

TriTrypDB ID	TriTrypDB description	Average EUPC ≥ 2
***Li***p47		
LinJ.36.1420 LinJ.05.1200 LinJ.22.0200 LinJ.28.0730 LinJ.09.1060 LinJ.14.0700 LinJ.29.0520 LinJ.32.0380 LinJ.07.0840 LinJ.27.2660 LinJ.34.1620	**Valosin-containing protein, putative** protein phosphatase type 1 (PP1) regulator-like protein (SDS22)* **SEP domain containing protein, putative (p47/Shp1)** serine/threonine protein phosphatase catalytic subunit, putative (Glc7/PP1-B)* PUB domain containing protein, putative (PUB1)* fatty acid elongase, putative (ELO2 GNS1/SUR4 family; 5 TMs) ADF (actin depolymerization factor)/Cofilin root hair defective 3 GTP-binding protein (RHD3), putative protein phosphatase inhibitor (Ypi1, PP1 Protein phosphatase inhibitor)* hypothetical protein, conserved (2 TMs) TPPP/p25-alpha, putative (Tubulin Polymerization-Promoting Protein)	38.5 19.5 18.0 8.0 7.5 4.5 4.0 3.5 3.0 3.0 2.0
***Li***UFD1		
LinJ.36.1420 LinJ.05.1040 LinJ.36.2850 LinJ.36.4740 LinJ.24.1650 LinJ.35.1960 LinJ.36.6780 LinJ.15.1570 LinJ.19.1240 LinJ.25.1320 LinJ.03.0250 LinJ.05.1140 LinJ.28.1390 LinJ.35.4920 LinJ.14.0700 LinJ.29.2300 LinJ.18.0450 LinJ.12.0550	**Valosin-containing protein, putative** stomatin-like protein 2 (SLP-2, mitochondrial)* mitochondrial ATP-dependent zinc metallopeptidase, putative (AAA domain ATPase family; FtsH protease) (1 TM)* hypothetical protein, conserved* UBA/TS-N domain containing protein, putative* UBX domain containing protein, putative (FAS-associated factor 2; FAF2)* ubiquitin fusion degradation protein, putative (UFD1)* hypothetical protein, conserved* peroxin 12 (PEX12), putative (RING/U-box) NPL4 family, putative* hypothetical protein, conserved (1 TM)* V-type proton ATPase subunit D, putative phenylalanine-4-hydroxylase (signal peptide) mitochondrial import receptor subunit ATOM40, putative fatty acid elongase, putative (ELO2 GNS1/SUR4 family; 5 TMs) hypothetical protein-conserved (signal peptide, 1 TM) serine carboxypeptidase (CBP1)-putative (mitochondrial, 1 TM) hypothetical protein-conserved (alkaline phosphatase like superfamily, 1 TM)	50.5 25.5 22.5 16.5 15.5 14.5 14.5 12.5 9.0 8.5 4.5 4.5 3.5 3.0 2.5 2.5 2.5 2.5
***Li***FAF2		
LinJ.36.1420 LinJ.05.1040 LinJ.36.2850 LinJ.35.1960 LinJ.24.1650 LinJ.15.1570 LinJ.36.4740 LinJ.05.1140 LinJ.17.0230 LinJ.07.0450 LinJ.14.0700 LinJ.25.2330 LinJ.14.0680 LinJ.32.0380 LinJ.14.0760 LinJ.33.1130 LinJ.36.5360 LinJ.35.4920 LinJ.29.0520 LinJ.34.4180 LinJ.35.3740 LinJ.26.1610 LinJ.34.1620 LinJ.25.2400	**Valosin-containing protein, putative** stomatin-like protein 2 (SLP-2, mitochondrial)* mitochondrial ATP-dependent zinc metallopeptidase, putative (AAA domain ATPase family; FtsH protease) (1 TM)* UBX domain containing protein, putative (FAF2)* UBA/TS-N domain containing protein, putative* hypothetical protein, conserved* hypothetical protein, conserved* V-type proton ATPase subunit D, putative tetratricopeptide repeat, putative (ER membrane protein complex subunit 2)* uncharacterized protein family (UPF0172) (ER membrane protein complex sub. 8)* fatty acid elongase, putative (ELO2 GNS1/SUR4 family; 5 TMs) glycosome import protein, putative (E3 ubiquitin-protein ligase, PEX2) fatty acid elongase, putative (ELO1 GNS1/SUR4 family; 6 TMs) root hair defective 3 GTP-binding protein (RHD3), putative fatty acid elongase, putative (ELO3 GNS1/SUR4 family; 5 TMs) hypothetical protein-conserved (1 TM) hypothetical protein-conserved mitochondrial import receptor subunit ATOM40, putative ADF (actin depolymerization factor)/Cofilin cell differentiation protein-like protein (Rcd1 homolog) peroxisomal biogenesis factor 11 (PEX11), putative 40S ribosomal protein S33, putative TPPP/p25-alpha, putative (Tubulin Polymerization-Promoting Protein) PEX10, PEX2 / PEX12 amino terminal region/ RING/U-box containing protein	19 25 19 17.5 13.5 11 8.0 8.0 7.5 5.0 5.0 5.0 3.5 3.0 3.0 3.0 2.5 2.5 2.5 2.5 2.5 2.0 2.0 2.0
***Li***PUB1		
LinJ.36.1420 LinJ.09.1060 LinJ.32.0380 LinJ.14.0700 LinJ.33.1130 LinJ.36.6780 LinJ.35.4920 LinJ.34.3530 LinJ.26.1610 LinJ.34.1620	**Valosin-containing protein, putative** PUB domain containing protein, putative (PUB1)* root hair defective 3 GTP-binding protein (RHD3), putative fatty acid elongase, putative (ELO2 GNS1/SUR4 family; 5 TMs) hypothetical protein, unknown function ubiquitin fusion degradation protein, putative (UFD1)* mitochondrial import receptor subunit ATOM40, putative serine palmitoyltransferase-like protein (long chain base biosynthesis protein) 40S ribosomal protein S33, putative TPPP/p25-alpha, putative (Tubulin Polymerization-Promoting Protein)	24.5 14.5 3.0 3.0 3.0 2.0 2.0 2.0 2.0 2.0

Proteins in bold represent the core *Li*VCP cofactors. Proteins indicated with an asterisk (*) are also found in *Li*VCP immunoprecipitates. TM: transmembrane helix. Average of exclusive unique peptide count (EUPC) is from seven independent IP experiments.

**Figure 3:**
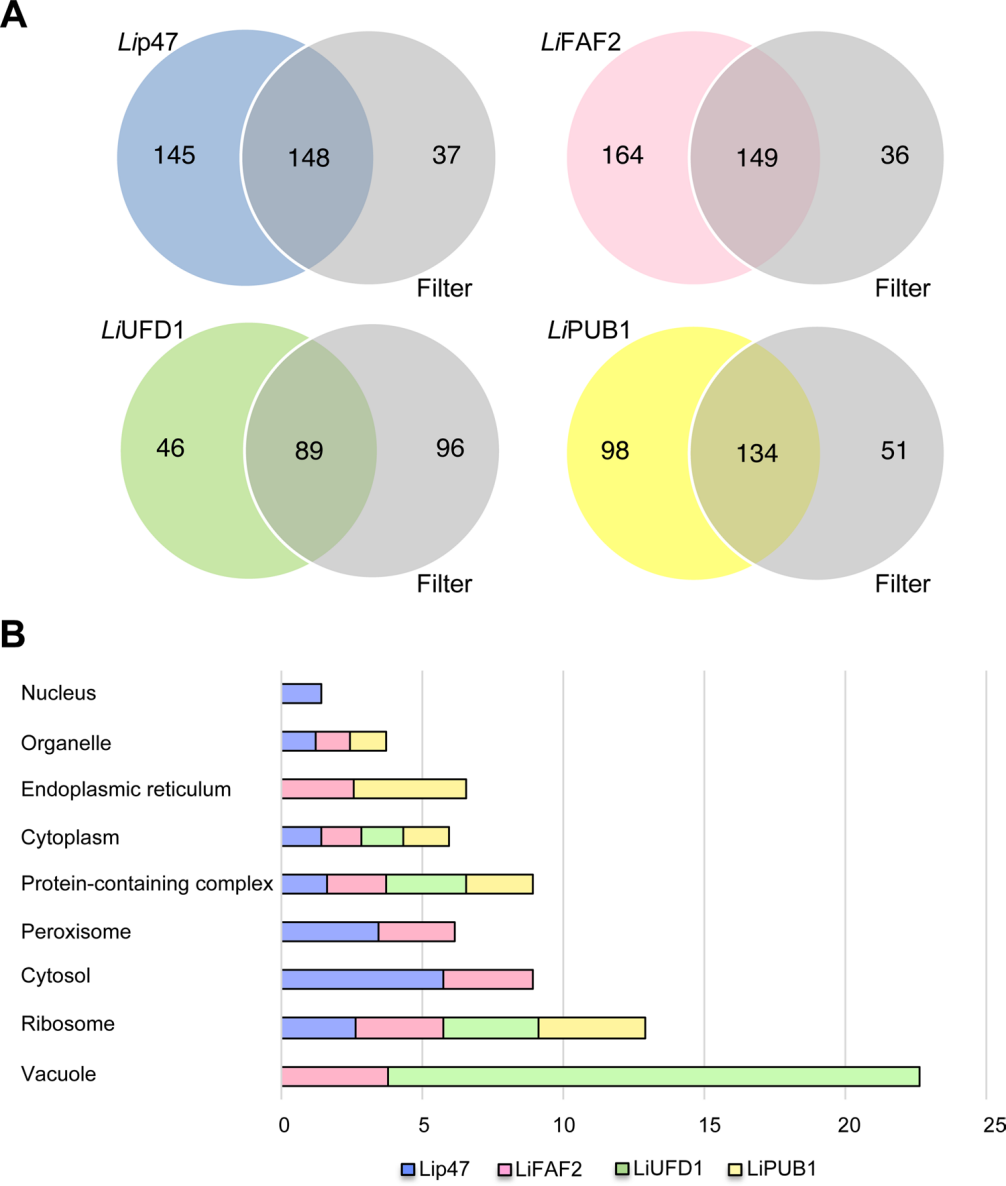
Network proteomics to gain novel insights into *Li*VCP cofactor complexes. (**A**) Venn diagrams demonstrating the number of proteins identified by mass spectrometry in *Li*p47, *Li*FAF2, *Li*UFD1 and *Li*PUB1 co-immunoprecipitates of two independent experiments for each cofactor (see Methods) after applying a filter of five unrelated HA-tagged proteins (average of EUPC = 3.6 on 5 unrelated proteins; see Supplementary Table S2 for details) to reduce non-specific interactions. (**B**) Gene ontology–cellular component (GO-CC) analysis of proteins identified in (A) for each *Li*VCP cofactor: *Li*p47 (145), *Li*FAF2 (164), *Li*UFD1 (46), and *Li*PUB1 (98) according to their terms listed on TriTrypDB. The same stringent filter (grey in A) was applied for each cofactor (see Supplementary Table S2 for details). Gene Ontology biological process and molecular function can be seen in Supplementary Fig. S19.

As done for *Li*VCP, we carried out IP-MS/MS studies with five unrelated proteins to VCP complexes (average EUPC > 3.6; *Li*VCP threshold) to filter out non-specific *Li*VCP cofactor partners (Fig. 3A and Supplementary Table S2). The remaining proteins that co-immunoprecipitated with *Li*p47 (145), or *Li*FAF2 (164), or *Li*UFD1 (46) and or *Li*PUB1 (98) can be considered as putative cofactor interacting partners (Fig. 3A, Table 1 and Supplementary Table S2).

*Li*p47 co-immunoprecipitated *Li*PUB1, the serine/threonine protein phosphatase 1 (PP1) catalytic subunit beta (LinJ.28.0730), a PP1 regulator-like protein that is homologous to the yeast SDS22 protein (LinJ.05.1200), and the PP1 phosphatase inhibitor that is homologous to the yeast Ypi1 protein (LinJ.07.0840) (Table 1). In *Saccharomyces cerevisiae,* these three proteins form a ternary complex that is important for the nuclear localization of PP1 and whose assembly and quality control requires Cdc48 and its adaptor p47^38^.

As reported in other eukaryotes^34^, the *Leishmania Li*UFD1 protein complexes with NPL4 to form the heterodimer cofactor UFD1-NPL4 but also associates with *Li*FAF2 (Table 1). Complex formation between VCP-NPL4-UFD1, FAF1 and polyubiquitinated proteins was shown previously to promote ER-associated degradation^39^. Other proteins only associated with the *Li*UFD1 proteome include a mitochondrial serine carboxypeptidase (CBP1; LinJ.18.0450), a phenylalanine-4-hydroxylase (LinJ.28.1390), peroxin PEX12 (LinJ.19.1240) and three hypothetical conserved proteins (LinJ.03.0250, LinJ.29.2300, LinJ.12.0550) (Table 1 and Supplementary Table S2). Interestingly, the hypothetical protein LinJ.03.0250 was also found in *Li*VCP immunoprecipitates (Fig. 1C), and some of the other *Li*UFD1 interacting proteins have homologs known to be part of the VCP network in other systems (see Discussion).

Proteins solely detected in *Li*FAF2 pull-down include a tetratricopeptide repeat containing protein of the ER membrane protein complex (LinJ.17.0230) also found in *Li*VCP immunoprecipitates (Fig. 1C), several peroxisomal/glycosomal proteins that are homologs of the peroxins PEX2 (LinJ.25.2330), PEX10 (LinJ.25.2400) and PEX11 (LinJ.35.3740), two fatty acid elongases (ELO1: LinJ.14.0680 and ELO3: LinJ.14.0760), a cell differentiation protein-like (LinJ.34.4180), and hypothetical protein LinJ.36.5360 (Table 1).

The *Li*PUB1 cofactor co-immunoprecipitated a serine palmitoyltransferase 1-like protein (LinJ.34.3530; SPT1), also found in the *Li*UFD1 proteome (Table 1). Serine palmitoyltransferase catalyzes the first and rate-limiting step in sphingolipid (ceramide) biosynthesis and has been associated with the resistance to heat stress and apoptosis^40,41^.

Interestingly, several *Li*VCP cofactor-associated proteins are common to more than two cofactors (see Table 1). For example, *Li*p47, *Li*FAF2 and *Li*PUB1 co-immunoprecipitated the tubulin polymerization-promoting protein TPPP/p25-alpha (LinJ.34.1620) that stabilizes microtubules^42^ and also protects against abnormal forms of prion proteins^43^, as well as the root hair defective 3 GTP-binding protein (RHD3) (LinJ.32.0380) that is analogous to the mammalian atlastin GTPases involved in shaping ER tubules^44^. An ADF/cofilin factor (LinJ.29.0520) of the family of actin remodeling proteins^45^ co-immunoprecipitated with both *Li*p47 and *Li*FAF2. The ribosomal protein S33 (LinJ.26.1610) and the hypothetical protein LinJ.33.1130 co-immunoprecipitated with *Li*FAF2 and *Li*PUB1. The V-type proton ATPase subunit D (LinJ.05.1140), a multi-subunit membrane protein complex that is evolutionarily related to F-type adenosine triphosphate synthases and A-ATP synthases^46^, co-immunoprecipitated with *Li*p47 and *Li*UFD1. The *Li*UFD1, *Li*FAF2 and *Li*PUB1 cofactors co-immunoprecipitated the trypanosomatid functional analog of Tom40 (ATOM40; LinJ.35.4920), which is the central pore of the TOM complex involved in the import of mitochondrial proteins^47^. Finally, all *Li*VCP core cofactors co-immunoprecipitated a fatty acid elongase (ELO2) (LinJ.14.0700).

### General features of the *Li*VCP core cofactors

Proteins specifically co-immunoprecipitated with *Li*UFD1, *Li*p47, *Li*FAF2, or *Li*PUB1 were submitted to Gene Ontology – cellular component (GO-CC) enrichment analysis using the tool provided by TriTrypDB (Fig. 3B and Supplementary Table S3). An enrichment of a 19-fold for vacuolar proteins was seen in the *Li*UFD1 proteome. Nuclear proteins were exclusively enriched within the *Li*p47 proteome and endoplasmic reticulum-related proteins were enriched only within the *Li*FAF2 and *Li*PUB1 proteomes.

Molecular function (MF) and biological process (BP) analyses were also done aside comparison with the *Li*VCP proteome (Supplementary Table S3). Globally, the molecular function analysis predicted proteins with translation factor activity and structural constituents of the ribosome, RNA-binding activity, structural molecule activity and peptidase activity. ATPase and ligase activities were exclusively enriched within the *Li*FAF2 proteome while transmembrane transporter activity was only associated with the *Li*UFD1 proteome.

The biological process analysis showed that cofactor-associated proteins were mostly involved in translation, protein targeting and folding, and several metabolic processes. Protein folding and tRNA metabolic processes were found only associated with the *Li*FAF2 proteome while protein targeting and mitotic cell cycle were enriched in the *Li*PUB1 proteome (Supplementary Table S3). Overall, Gene Ontology analysis of each proteome indicated specific compartment-association for each cofactor.

### Subcellular localization of the *Li*VCP core cofactors

To determine the subcellular localization of the core *Li*VCP cofactors, we carried out digitonin fractionation and indirect immunofluorescence studies. As a central player to the endoplasmic reticulum (ER)-associated protein degradation^19,48^ and the translocation of damaged mitochondrial proteins from the outer mitochondrial membrane (OMM) into the cytosol^20,49^, VCP has been found associated with both ER and OMM fractions, similarly to what we have described previously for the *Leishmania Li*VCP^10^.

**Figure 4:**
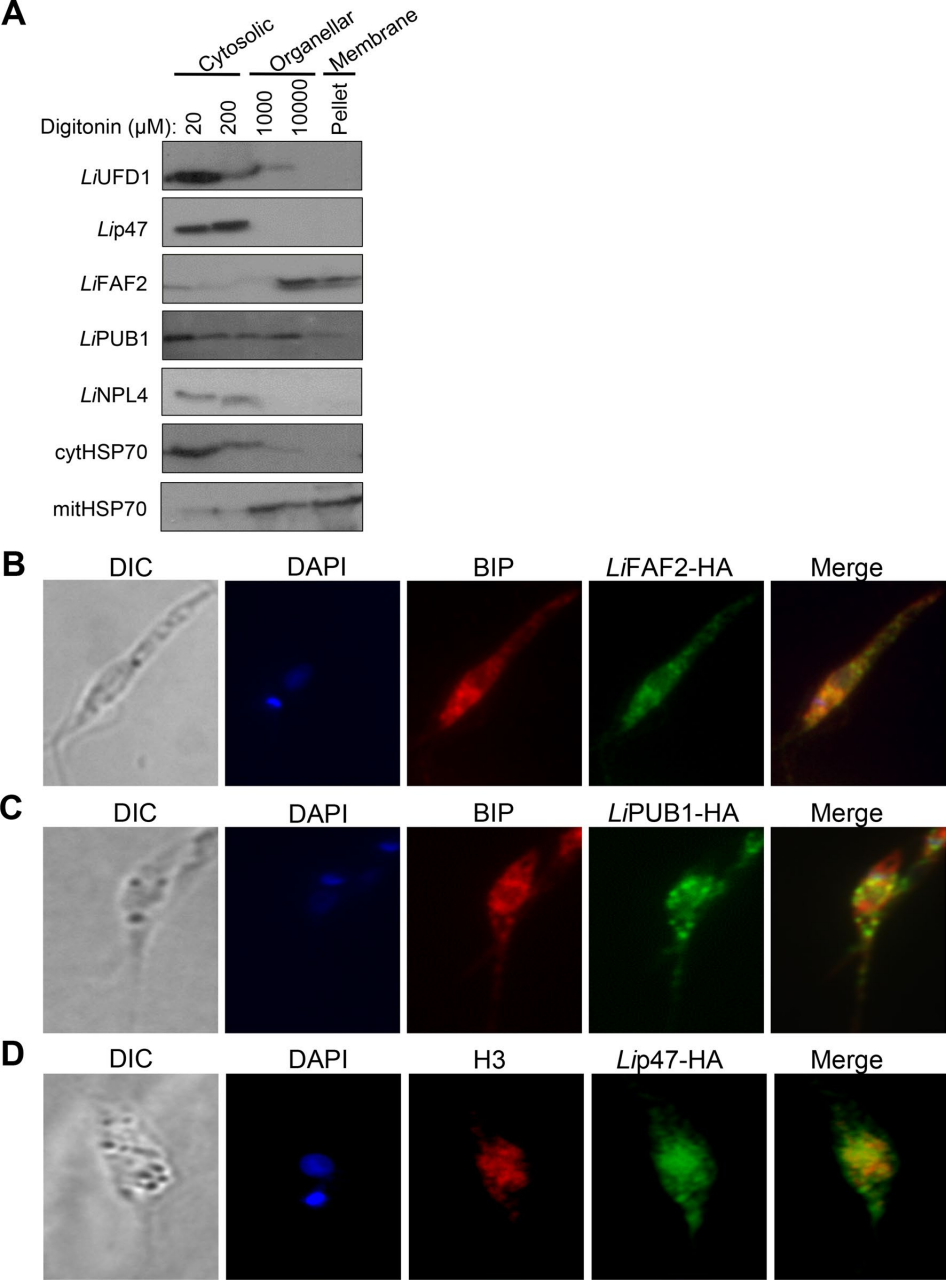
Subcellular localization of *Li*VCP cofactors. (**A**) Western blot of *L. infantum* digitonin‐fractionated promastigotes (20 μM-10 mM) using the anti‐HA antibody to detect the C- or N-terminally HA-tagged *Li*UFD1, *Li*NPL4, *Li*p47, *Li*FAF2 and *Li*PUB1 proteins ectopically expressed in *Leishmania*. The 20 μM to 200 μM digitonin fractions are enriched with cytosolic proteins, the 1 mM and 10 mM fractions contain mostly organellar proteins, and the pellet fraction contains membrane‐associated proteins. Antibodies against the cytosolic or mitochondrial HSP70 were used as controls. The uncropped blots are shown in Supplementary Fig. S20 (panels E-I). Immunofluorescence studies showing the localization of C- or N-terminally HA-tagged *Li*FAF2 (**B**), *Li*PUB1 (**C**) and *Li*p47 (**D**) (in green). An anti‐HA antibody was used as primary antibody followed by Alexa Fluor 488 anti-mouse as secondary antibody. Nuclear and kinetoplast DNA was stained with DAPI (blue). Endoplasmic reticulum (ER) putative co-localization was assessed using an anti‐BiP antibody (red in B and C) as an ER marker. An Alexa Fluor 555 anti-rabbit was used as secondary antibody for BiP. Putative nuclear co-localization for *Li*p47 was assessed using an anti‐histone H3 (H3) antibody (red in D).

First, we treated *Leishmania* parasites with increasing concentrations of digitonin (20 μM-10 mM) and carried out western blotting on the different cellular sub-fractions using an anti-HA antibody (all cofactors were HA-epitope tagged). Our results indicate an association of *Li*FAF2 and partly of *Li*PUB1 proteins with the organellar fraction and an enrichment of *Li*UFD1, *Li*p47 and *Li*NPL4 proteins with the cytosolic fractions (Fig. 4A). Immunofluorescence studies using an antibody directed against the ER BiP protein confirmed that *Li*FAF2 and partly *Li*PUB1 co-localize to endoplasmic reticulum (Fig. 4B, 4C). No co-localization was found with the mitochondrion (data not shown). Immunofluorescence studies to detect *Li*p47 demonstrated a partial co-localization with the histone H3 nuclear marker (Fig. 4D), as also suggested by GO analysis (Fig. 3B).

### Predicted molecular interactions between the conserved domains of *Li*VCP cofactors and the *Leishmania* VCP protein based on 3D homology modeling and protein–protein docking

**Figure 5:**
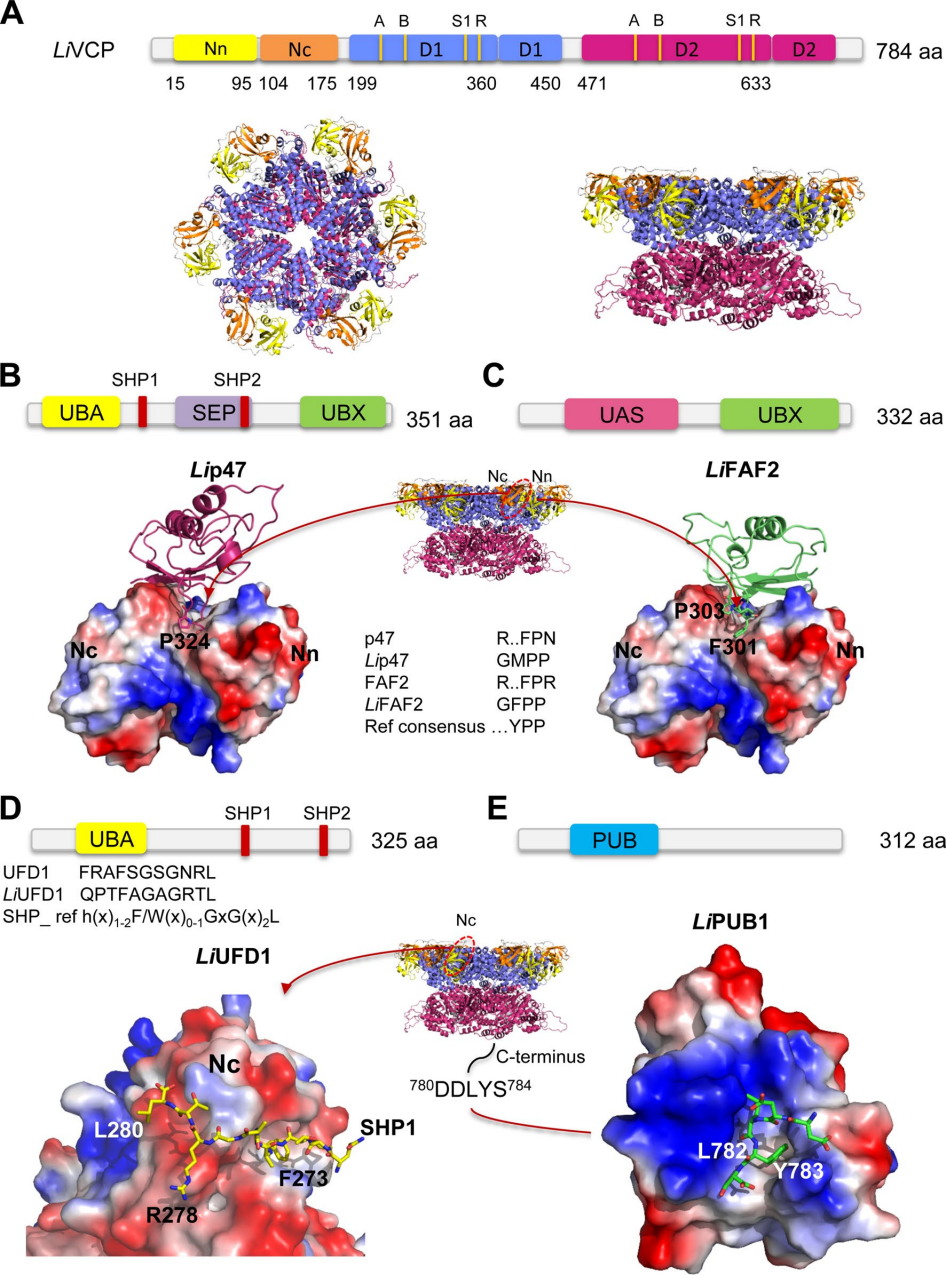
3D homology modeling of the major *Li*VCP cofactors and their docking prediction into the *L. infantum Li*VCP protein. (**A**) Top, domain architecture of *Li*VCP. The N domain is subdivided into subdomains Nn (colored in yellow) and Nc (colored in orange) and the two ATPase domains are in blue (D1) and fuchsia (D2), respectively. The Walker A (A) and Walker B (B) motifs as well as the Sensor 1 (S1) residues and arginine (R) fingers are indicated. Bottom, top (left) and side views (right) of the *Li*VCP hexamer 3D structure model built by homology with the *H. sapiens* p97 hexamer (PDBid entry 5C18). (**B**) Top, domain composition of *Li*p47. Bottom, electrostatic potential surface of *Li*VCP (colored according to hydrophobicity) with stick representation of the UBX GMPP binding motif of *Li*p47. Key interactions are shown. (**C**) Top, domain composition of *Li*FAF2. Bottom, electrostatic potential surface of *Li*VCP with stick representation of the UBX GFPP binding motif of *Li*FAF2. Middle panel between B and C shows molecular surface of *Li*VCP with the UBX binding site indicated (Nn-Nc pocket). (**D**) Top, domain composition of *Li*UFD1. Bottom, electrostatic potential surface of *Li*VCP with stick representation of the SHP1 binding site QPTFAGAGRTL. (**E**) Top, domain composition of *Li*PUB1. Bottom, electrostatic potential surface of *Li*PUB1 with a stick representation of the C-terminus of *Li*VCP ^780^DDLYS^784^. Middle panel between D and E shows molecular surface of *Li*VCP with the SHP binding site (Nc) and C-terminus tail indicated. 3D homology models of *Li*UFD1, *Li*p47, *Li*FAF2 and *Li*PUB1 were respectively built by homology based on the templates with pdb entries: 5C1B_V, 1S3S_H, 2MX2 and 2HPL, respectively using the modeling software MODELLER^80^. For further details, see Supplementary Figs. S9, S11, S13 and S18. UBA (Ubiquitin-associated),SEP (Shp, eyes-closed, p47),SHP (BS1, binding segment 1); UBX (Ubiquitin regulatory X); UAS, domain of unknown function found in FAF2 and other proteins; PUB (PNGase/UBA or UBX) containing proteins. Further information about the respective domains can be found at https://www.ebi.ac.uk/interpro/.

It has been shown previously that the VCP/p97/Cdc48 hexamer can bind to its various cofactors through the UBX or PUB domains or a ubiquitin-like fold or through one of the linear binding motifs such as SHP^32^. The *L. infantum Li*VCP encodes a protein of 784 amino acids that is highly conserved among its eukaryotic orthologs (75% aa identity with the human VCP/p97 protein)^10^ and harbors all the characteristic domains of VCP orthologs (Fig. 5A, top). Based on sequence similarity and conservation to mammalian VCP cofactor orthologs (Supplementary Fig. S1) and on the EUPC for each cofactor in *Li*VCP immunoprecipitates (Fig. 1), we selected *Li*p47, *Li*UFD1, *Li*NPL4, *Li*FAF2, and *Li*PUB1 as the core *Li*VCP cofactors for further investigation. Multiple sequence alignments of these *Li*VCP cofactors along with mammalian, yeast, trypanosomatid and non-trypanosomatid species helped us to generate phylogenetic trees revealing that *Li*p47, *Li*FAF2, *Li*UFD1, *Li*NPL4 and *Li*PUB1 are evolutionary distant from other eukaryotes but they harbor binding modules/motifs that are conserved and could represent specific domains for interaction with *Li*VCP (Supplementary Figs. S3-S7).

To investigate whether *Li*VCP-cofactor interactions identified by immunoprecipitation studies take place within the previously predicted binding sites in the hexameric VCP protein^30,32^ and the conserved binding modules in *Li*p47, *Li*FAF2, *Li*UFD1, *Li*NPL4 and *Li*PUB1, we constructed 3D models by homology modeling and a protein–protein docking approach. 3D homology models are helpful in refining protein–protein interaction predictions that have been based on a sequence match alone as interface binding is more directly determined by the structure of the binding site rather than its sequence. The *Li*VCP homohexamer 3D molecular structure was obtained through homology modeling by superposition with the *H. sapiens* p97 homohexamer (PDBid: 5C18) template (Fig. 5A, bottom).

The *Li*p47 protein presents the same domain composition (UBA, SEP and UBX) than its human ortholog (Fig. 5B, upper). Similarly to the human p47, *Li*p47 harbors the sequence GMPP in the UBX domain (Supplementary Fig. S8) that docks in silico into the Nn-Nc pocket of *Li*VCP (Fig. 5B and Supplementary Fig. S9)^50^. Although *Li*p47 shares only 25% amino acid identity with its human ortholog, it has 41% sequence similarity and 73% sequence coverage and contains the two SHP motifs (SHP1 and SHP2) (Supplementary Fig. S8) that together with the UBX domain are known to be key for the interaction with VCP^31^. The SHP site is typically characterized by the consensus sequence FxGxGx_2_h that was recently summarized as h(x)_1−2_F/W(x)_0−1_GxGx_2_L (h, hydrophobic residue; x, any amino acid)^32^ and in *Li*p47, SHP1 and SHP2 sites, FYGRGQRL and FQGHGHRL, respectively are conserved (Supplementary Fig. S8). In addition, the Ramachandran plot showed that the amino acid residues of the *Li*p47 3D homology model are found in most favoured (87%) and additional allowed regions (8.7%) (Supplementary Table S4), which makes structure predictions reliable.

The *Li*FAF2 protein interacts with *Li*VCP through the GFPP motif in its UBX C-terminal domain as predicted by 3D homology modeling (Fig. 5C, Supplementary Fig. S10) and protein–protein docking upon superposition of the available structure of the human ^40^GYPP^43^ motif in the ovarian tumor domain-containing protein 1 (OTU1) and the homologous part within the *Leishmania* FAF2 protein (Supplementary Fig. S11). Surprisingly, *Li*FAF2 lacks the ubiquitin-associated (UBA) domain that is present in the human FAF1 and FAF2 proteins and is fundamental for interacting with ubiquitinated substrates^51^.

UFD1 is known to assemble with NPL4 to form the heterodimer UFD1/NPL4 (U/N), one of the most important p97/VCP cofactors, that recognizes polyubiquitinated substrates and together with VCP extracts them from organellar membranes or macromolecular complexes^52,53^. U/N binds to the N-terminal domain of VCP via the ubiquitin-like domain (UBL) of NPL4 and the SHP box of UFD1^32^. The *Leishmania Li*UFD1 protein also contains the UBA domain and an UT6 region that accommodates the sequences QPTFAGAGRTL and RLKALGGGGRS corresponding to the SHP1 and SHP2 sites, respectively (Fig. 5D, top and Supplementary Fig. S12). 3D homology modeling predicts binding of *Li*UFD1 to *Li*VCP via its SHP1 site (Fig. 5D, bottom). Furthermore, docking essays propose that the SHP1 motif of *Li*UFD1 (reversed comparatively to the human SHP1) interacts with the same surface as in its human ortholog using amino acid residues Phe273, Arg278 and Leu280 (Phe228, Arg234 and Leu235 in human Ufd1) (Fig. 5D bottom and Supplementary Fig. S13).

In contrast to its eukaryotic orthologs, the *Leishmania* NPL4 lacks the N-terminal ubiquitin regulatory X (UBX)-like domain bound to the N-domain of Cdc48 hexamer, as well as the two Zn^2+^-finger domains important for anchoring the adjacent Mpr1/Pad1 N-terminal (MPN) domain to the top of the D1 ATPase ring^34^. Interestingly, protein–protein docking simulations showed that despite the absence of the UBX-like domain, *Li*NPL4 seems to interact with the N-terminus of *Li*VCP through its C-terminal GMPP motif (aa 350–353) (Supplementary Figs. S14, S15). Similarly to other eukaryotes^34^, the U/N heterodimer in *Leishmania* also recognizes polyubiquitinated substrates as shown by *Li*NPL4 and *Li*UFD1 IPs followed by western blotting (Supplementary Fig. S16).

A representative member of the PUB (PNGase/UBA or UBX-containing) domain proteins in *Leishmania*, *Li*PUB1, which has no homolog in humans, contains a conserved region (Supplementary Fig. S17) that is predicted by 3D homology modeling (Fig. 5E) and protein docking (Supplementary Fig. S18) to interact with the last five amino acids ^780^DDLYS^784^ of the *Li*VCP C-terminus, similarly to other PUB domain proteins ^54^.

Altogether, despite the evolutionary distance of *Li*VCP cofactors from their mammalian orthologs, in silico models based on structural data from the human orthologs using 3D homology modeling and protein–protein docking indicated that the conserved binding modules of *Leishmania* VCP core cofactors (except *Li*NPL4) interact with specific binding sites in the hexameric *Li*VCP, similarly to their mammalian orthologs.

### The first trypanosomatid VCP interaction network

**Figure 6:**
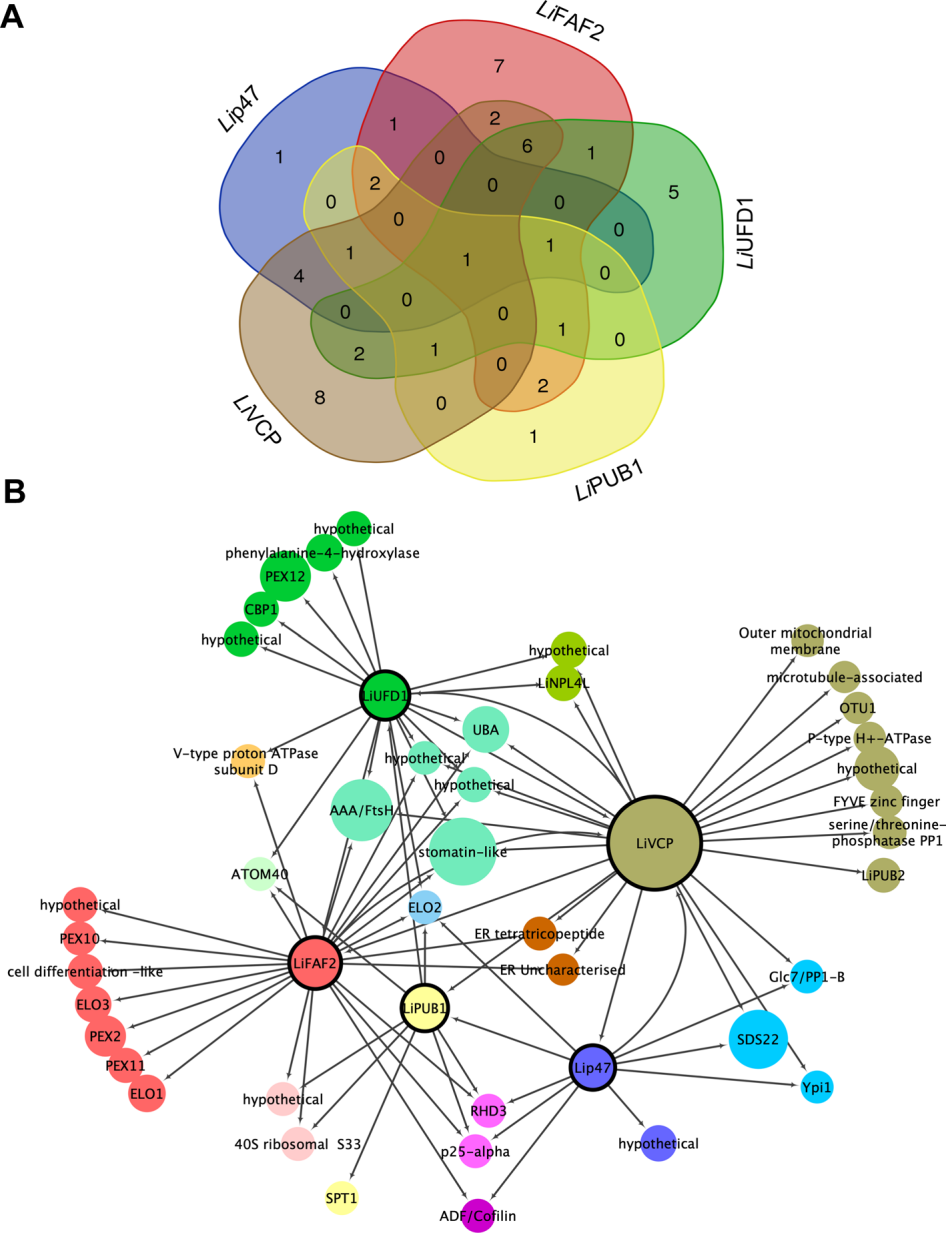
The *Leishmania* VCP protein interaction network. **(A)** A Venn diagram representing the number of proteins identified by mass spectrometry in independent immunoprecipitates of *Li*VCP and its key cofactors *Li*UFD1, *Li*p47, *Li*FAF2 and *Li*PUB1. For high stringency filtering and network clarity, only proteins absent in IPs with HA-tagged unrelated proteins (EUPC = 0.0 on five unrelated experiments) were considered for this analysis. The intersections of list of elements were calculated with the Venn tool at https://bioinformatics.psb.ugent.be/webtools/Venn/. For the list of proteins used and their intersection, see Supplementary Table S5. **(B)** Integrated interaction map of the *Li*VCP network using the data generated in this work showed as in (A). For clarity, protein names were simplified. Filled color circles for *Li*VCP and its cofactors are represented as in (A). Bigger circles represent higher average of EUPC in *Li*VCP IP experiments. The proteins *Li*UFD1, *Li*p47, *Li*FAF2, *Li*PUB1 and *Li*VCP were used as bait (see Methods) and are represented with bold border circles. The direction of interactions is represented with a target arrow shape. Spring-embedded layout was applied on Cytoscape 3.5.1.

The resulting datasets from *Li*VCP, *Li*p47, *Li*UFD1, *Li*FAF2 and *Li*PUB1 IP-MS/MS studies were mined to identify interacting partners that are specific to each *Li*VCP cofactor as well as partners that are shared among multiple cofactors and *Li*VCP complexes (Fig. 6A) and allowed us to build the first *Li*VCP interaction network (Fig. 6B). To decrease the number of false positives, we excluded proteins that were co-immunoprecipitated with unrelated HA-tagged proteins (average of EUPC = 0.0). From the total number of proteins co-immunoprecipitated with *Li*p47, *Li*UFD1, *Li*FAF2, and *Li*PUB1 (Fig. 3A) only 10, 17, 23 and 9, respectively together with 24 proteins specifically associated to *Li*VCP (Fig. 1^,^Table 1 and Supplementary Table S2) were considered to build the topology of *Li*VCP network (83 proteins in total, 47 shown in Fig. 6B). The list of proteins and their intersections are detailed in Supplementary Table S5 whereas the complete list of interacting proteins is presented in Supplementary Table S2.

In addition to the known cofactors, eight proteins were exclusively associated with the *Li*VCP proteome and might also be considered as cofactors of the *Leishmania* VCP protein. These include another PUB domain protein (PUB2) (LinJ.11.0920), an OTU1 (ovarian tumor domain-containing protein 1-like cysteine protease (LinJ.36.6280) that is homologous to the *S. cerevisiae* deubiquitylation enzyme OTU1 shown to interact with VCP^55^, a protein present in the outer mitochondrial membrane (LinJ.29.2220), a P-type H + -ATPase (LinJ.18.1510), a microtubule-associated protein (LinJ.26.1950), a member of the FYVE zinc finger proteins (LinJ.36.2570) that serve as regulators of endocytic membrane trafficking and receptor signaling^56^, a type 1 protein serine/threonine phosphatase (PP1) (LinJ.34.0900) and a *Leishmania* specific hypothetical protein (LinJ.36.5080) (Fig. 6B and Supplementary Table S5).

Our analysis revealed that the *Li*UFD1 and *Li*FAF2 proteomes share five proteins in common with *Li*VCP. These include the mitochondrial stomatin-like protein 2 (SPL-2) (EUPC > 20), the membrane-integrated mitochondrial AAA/FtsH protease^57^, an UBA domain containing protein (LinJ.24.1650), and hypothetical conserved proteins LinJ.15.1570 and LinJ.36.4740 (Table 1, Fig. 6B). *Li*VCP and *Li*p47 mutually associate with the serine/threonine protein PP1 phosphatase catalytic subunit, the PP1 phosphatase inhibitor Ypi1, and the SDS22 (EUPC = 20) PP1 regulator-like protein (Table 1, Fig. 6B and Supplementary Table S5). *Li*VCP and *Li*FAF2 mutually co-immunoprecipitate the tetratricopeptide repeat protein (LinJ.17.0230) and an uncharacterized protein family (LinJ.07.0450), both subunits of the ER membrane protein complex (Table 1, Fig. 6B and Supplementary Table S5). *Li*VCP and *Li*UFD1 mutually interact with the hypothetical protein LinJ.03.0250 (Table 1, Fig. 6B and Supplementary Table S5). Specific interactions between cofactor-associated partners that do not involve an association with *Li*VCP are described in detail in the ‘network proteomics’ section (see also Table 1 and Fig. 6B).

Overall, using combined datasets from multiple IP/MS–MS studies for *Li*VCP and its main cofactors, we unveiled the first *Leishmania Li*VCP interactome. Many of the *Li*VCP interactors have homologs that are known to interact with VCP in other eukaryotic systems but others do not and may be relevant to study further as part of distinct *Leishmania* VCP complexes.

## Discussion

In this study, we have characterized the valosin‐containing protein (VCP) interaction network, the first in parasitic protozoa. We have employed a series of co-immunoprecipitation and mass spectrometry analyses coupled with in silico models that allowed us to identify the *Leishmania* VCP proteome with its major cofactors and their interacting partners, as well as, to predict molecular interactions between conserved domains within these cofactors and specific binding sites in the hexameric *Li*VCP. Our data support several similarities but also important differences between the *Leishmania* VCP protein network and VCP complexes characterized in other eukaryotes.

Most of the known classes of VCP cofactors in other eukaryotes have orthologs in *Leishmania*. However, cofactors harboring the VIM (VCP-interacting motif)/VBM (VCP-binding motif) motifs^58^ were not found in any of our immunoprecipitation experiments, and with the exception of the ERAD-associated E3 ubiquitin-protein ligase HRD1 (LinJ.15.1460) no homologs of this class of proteins were depicted in the *Leishmania* genome. While *Li*VCP is highly conserved among different eukaryotic organisms and harbors all the characteristic domains of the human p97/VCP ortholog^10^, most of the major *Li*VCP cofactors are phylogenetically distant from their eukaryotic orthologs. However, despite their evolutionary distance from yeast and mammalian orthologs, in silico models based on 3D homology modeling and protein–protein docking indicated that the conserved binding modules of *Leishmania* VCP cofactors interact with specific binding sites in the hexameric *Li*VCP, similarly to their eukaryotic orthologs. This is the case for *Li*p47 and *Li*FAF2 that bind the Nn-Nc pocket of *Li*VCP through the sequence GMPP/GFPP in their UBX domain. Surprisingly, *Li*FAF2 lacks the UBA domain and theoretically should have lost the ability of binding ubiquitinated substrates^59^. Similarly to its yeast ortrholog^34^, *Li*UFD1 interacts with the N-terminus of *Li*VCP through its conserved SHP motif. *Li*PUB1 while has no human homolog yet interacts with the C-terminal region of *Li*VCP, as described for the human VCP-PUB complex.

One of the important differences unveiled by our study concerns *Li*NPL4, which lacks the UBX-like domain and the two Zn^2+^-finger motifs shown to bind VCP. Indeed, recent studies on the structure of the Cdc48-Ufd1-Npl4 complex from the thermophilic fungus *Chaetomium thermophilum* demonstrated that Npl4 interacts with the Cdc48 N-terminal region through its UBX-like domain and uses the Zn^2+^-finger motifs to anchor its MPN domain to the top of the D1 ATPase ring^34^. On the other hand, Ufd1 interacts with Npl4 to form the Ufd1-Npl4 heterodimer through a short segment of its UT6 domain^34^ which is also conserved in the *Leishmania* Ufd1 ortholog. Our 3D homology modeling and docking experiments predicted binding of *Li*NPL4 to *Li*VCP through its C-terminus GMPP motif. All NPL4 orthologs of the genus *Leishmania*, *Leptomonas* and *Phytomonas* harbor the GMPP motif and a variant of this motif, GM-[EDS]-PP, is present in *Trypanosoma* species. This unique feature in Trypanosomatidae paves the way for the design of inhibitors specifically targeting NPL4 binding to VCP. Ufd1-Npl4 and p47 are substrate-recruiting cofactors shown to bind VCP in a mutually exclusive manner as they compete for the same binding modules^30,31^. This seems also to be the case in *Leishmania*. *Li*p47 was neither found in the *Li*UFD1 proteome nor *Li*UFD1 in the *Li*p47 pull-down. Moreover, they do not share common interacting partners, except for the V-type proton ATPase subunit D.

Our analysis led us to identify the first PUB domain proteins in *Leishmania* and to unveil *Li*PUB1 as one of the major *Li*VCP cofactors interacting with the C-terminal region of *Li*VCP. Furthermore, new prospects were opened with the yet non-studied PUB domain protein *Li*PUB2 identified here as a putative *Li*VCP cofactor. In addition, a third UBX- and PUB domain-containing protein (LinJ.36.0140; *Li*PUB3) was retrieved by searching the *L. infantum* genome database. PUB domain proteins known to bind to the C-terminus of the VCP hexamer have been associated with autophagy of damaged lysosomes^23^ but the physiological significance of these interactions remains to be determined. Here, we found that a serine palmitoyltransferase-like protein was associated solely with the *Li*PUB1 proteome. Interestingly, a homolog of this protein in yeast is required for resistance to heat shock and plays an essential role in the removal or refolding of denatured or aggregated cytoplasmic proteins^60^. We have reported recently that *Li*VCP is essential for the parasite survival under heat stress^10^, and PUB proteins with the help of a serine palmitoyltransferase may contribute to this process.

Interestingly, our study revealed new *Li*VCP interacting partners exclusively associated with the *Li*VCP proteome that might function as *Li*VCP cofactors. These include a PUB domain protein (*Li*PUB2), an outer mitochondrial membrane protein, a P-type H + -ATPase with homology to fungal and plant proton pumps^61^, a FYVE zinc-finger protein known to bind phosphatidylinositol 3-phosphate in membranes of endocytic vesicles that regulate membrane trafficking and receptor signaling^56^, a microtubule-associated protein, and several hypothetical conserved proteins that may be part of distinct VCP-complexes. *Li*VCP, and also *Li*p47, associates with the type 1 protein serine/threonine phosphatase (PP1) complex consisting of the PP1 catalytic subunit Glc7/PP1-B, the SDS22 regulator-like protein and the protein phosphatase PP1 inhibitor YPI1. In *S. cerevisiae,* nuclear localization of Glc7 requires Sds22 and Ypi1^62^. It has been shown recently that nuclear PP1 activity is positively regulated by the AAA-ATPase Cdc48 and its cofactor Shp1 (p47)^63^ to promote the assembly of the Glc7-Sds22-Ypi1 PP1 complex and to ensure its quality control^38^. Interestingly, here we found that *Li*p47 interacts with a large number of nuclear proteins and that is partly localized to the nucleus. From an evolutionary point of view, it is interesting that the interaction of *Li*VCP with *Li*p47 may regulate the assembly of the Glc7-Sds22-Ypi1 complex like is the case in yeast. *Li*VCP was found also associated, although not in all immunoprecipitates, with substrate-processing cofactors like the thioesterase ovarian tumor domain-containing protein 1 (OTU1), a deubiquitinating enzyme involved in ERAD through the interaction of its UBX-like (UBXL) domain with the N-terminus of Cdc48^64^. Although it is yet unclear how the polyubiquitinated substrates are released from the p97/VCP/Cdc48 complex and passed on to the proteasome^65^, OTU1 can be the deubiquitinase involved in this process as its UBXL domain interacts with VCP^55^. Although the *Leishmania* OTU1 ortholog does not harbor a well-defined UBXL domain, interestingly the ^39^GYPP^42^ loop of UBXL in *S. cerevisiae* shown not only to be critical for the interaction with VCP but also for its role in the ERAD pathway^55^ it is conserved in *Leishmania* (^49^GFPP^52^).

Another novel finding here is the association of the *Leishmania* VCP and also of its cofactors *Li*FAF2 and *Li*UFD1 with a stomatin-like protein 2 (SLP-2). Stomatins are members of the SPFH (stomatin, prohibitin, flotillin, HflC/K) superfamily that localize to the mitochondrial inner membrane scaffolding for the spatial organization of inner membrane proteases regulating mitochondrial dynamics, quality control, and cell survival^66^. Accordingly, our data support the association of *Li*UFD1 with transmembrane transport and peptidase activity proteins. It is possible that *Li*VCP associates with SLP-2 through UFD1, which also interacts with FAF2. Also present in the *Li*VCP proteome and associated with both *Li*FAF2 and *Li*UFD1 cofactors is the mitochondrial ATP-dependent AAA + protease FtsH whose proteolytic and chaperone-like activity is crucial to the protein degradation protein quality control of mitochondrial and chloroplast membranes^67^. This suggests a new model for membrane protein degradation mediated by ATP-dependent proteolytic systems^57^.

Our data support a subcellular compartmentalization for the key *Li*VCP cofactors. *Li*FAF2 and *Li*PUB1 are associated with organelles, *Li*FAF2 most likely with endoplasmic reticulum (ER), *Li*p47 is partially localized to the nucleus, *Li*UFD1 is possibly associated with cytosolic vacuoles, and NPL4 is enriched in the cytoplasm. Interestingly, *Li*FAF2 is associated with the peroxisomal biogenesis factors PEX2, PEX10 and PEX11. Similarly to other eukaryotes^68^, recent data in trypanosomatids support de novo biogenesis of peroxisomes (glycosomes) from the ER^69^. In higher eukaryotes PEX2, PEX10 and PEX12 are part of the peroxisomal E3 ubiquitin ligase complex required for pexophagy, a pathway to degrade ubiquitinated peroxisomes that involves an AAA ATPase complex with a striking similarity to p97^70^. Glycosome turnover in *Leishmania* is mediated by autophagy^71^ and the VCP/p97 quality control system, possibly through its FAF2 cofactor, may contribute to the control of glycosome homeostasis and turnover in these parasites. *Li*FAF2 also associates with two fatty acid elongases of the ELO GNS1/SUR4 family involved in the membrane-bound fatty acid chain elongation^72^. Interestingly, an ELO2 fatty acid elongase was found in the proteome of all four *Li*VCP cofactors (p47, UFD1 FAF2, PUB1). In trypanosomatids, the fatty acid elongation pathway occurs in the membrane of the ER^73^ and this could explain its association with components of the ERAD pathway.

In summary, this study allowed us to build the first VCP protein interaction network in trypanosomatids through the identification of known and novel interacting partners potentially associated with distinct VCP complexes. Our proteomics datasets identified biologically relevant functions for the *Leishmania* VCP cofactors and provided an important resource for further investigation of VCP function in several cellular processes related to protein quality control in these parasites.

## Methods

### Parasite strains, plasmid constructs and transfections

*Leishmania infantum* MHOM/MA/67/ITMAP-263 was used in this study. *L. infantum* promastigotes were cultured in SDM-79 medium supplemented with 10% heat-inactivated FCS (Multicell Wisent Inc., Canada) and 5 μg/ml hemin at pH 7.0 and 25 °C. The *Lip47* (LinJ.22.0200/LINF_220008200), *LiFAF2* (LinJ.35.1960/LINF_350024700), *LiUFD1* (LinJ.36.6780/LINF_360076400), *LiNPL4* (LinJ.25.1320/ LINF_250019000), and *LiPUB1* (LinJ.09.1060/LINF_090016300) genes were amplified and cloned into pSP72αZEOα. An HA epitope tag was added either at their N-or C-terminus. Primers used in this study are shown in Supplementary Table S6. The generated plasmids were independently transfected into *L. infantum* promastigotes. Purified plasmid DNA (10–20 μg, Qiagen Plasmid Mini Prep Kit, Toronto, Ontario, Canada) or linearized fragments (10–20 μg for the targeting cassette 5′flank‐αZEOα‐VCP^HA^‐3′flank from^10^ were transfected into *Leishmania* by electroporation as described previously^74^. Stable transfectants were selected and cultured with 0.6 mg/ml zeocin (Sigma).

### Protein lysate preparations and western blots

Western blots were performed following standard procedures. The anti-mouse HA tag monoclonal antibody (1:3,000; ABM), the anti-mouse HSP70 (cytosolic) antibody (1:400, Acris), the anti-rabbit HSP70 (mitochondrial) antibody (1:2000, kindly provided by Dr. Osvaldo de Melo Neto, Recife, Brazil), and the anti-mouse *T. brucei* (*Tb*)VCP antibody (1:5,000;^37^ kindly provided by Dr. James D. Bangs (Department of Microbiology & Immunology, Jacobs School of Medicine & Biomedical Sciences, University at Buffalo, USA) were used in this study. As a secondary antibody, we used the anti-mouse Horseradish peroxidase (HRP) (Cell Signalling) or the anti‐rabbit‐HRP antibody (GE Healthcare). Blots were visualized by chemoluminescence with Pierce ECL2 western blotting kit (Thermo Scientific). Digitonin fractionation was carried out in the presence of increasing concentrations of digitonin (20 μM to 10 mM) as described previously^75^ and each fraction was analysed by western blot.

### Immunofluorescence studies

Immunofluorescence studies were done as described previously^9,10^. The anti-mouse HA tag monoclonal antibody (1:1,000; ABM), the anti-mouse H3 Histone 3 antibody (1:500; ABM) and the rabbit polyclonal anti‐*T. brucei* BiP (kindly provided by Dr. JD Bangs) were used as a primary antibody followed by Alexa Fluor 488 anti-mouse and Alexa Fluor 555 anti-rabbit as secondary antibodies. Nucleus and kinetoplast DNA was stained with DAPI (blue). The cells were observed under a Nikon epifluorescence microscope with a 100X objective and oil immersion. Images acquisition was performed with ImagePro Plus software and ImageJ.

### Immunoprecipitation studies

Immunoprecipitation (IP) studies and mass spectrometry analysis were carried out as described previously^9^. Briefly, *L. infantum* promastigotes expressing HA-tagged protein were lysed, mixed with Pierce anti-HA magnetic beads (Thermo Scientific, Canada) and kept at -20 °C and subjected to mass spectrometry analysis or alternatively resolved in SDS-PAGE, excised, trypsin-digested, and analyzed by mass spectrometry.

### Sample preparation and LC–MS/MS analysis

Protein digestion and mass spectrometry analyses were performed by the Proteomics Platform of the CHU de Québec Research Center (Quebec, Qc, Canada) as described previously^76^. Briefly, bands of interest were extracted from gels, placed in 96-well plates and then washed with water. Tryptic digestion was performed on a liquid handling robot (MultiProbe, Perkin Elmer) according to manufacturer’s instructions using the protocol detailed in^77^ with some modifications as suggested in^78^*.* Proteins were reduced with 10 mM DTT and alkylated with 55 mM iodoacetamide. Iodoacetamide derivative of cysteine was specified as a fixed modification and oxidation of methionine and deamidation of Asparagine and Glutamine were specified as a variable modification. Two missed cleavages were allowed. Trypsin digestion was performed using 126 nM of modified porcine trypsin (Sequencing grade, Promega, Madison, WI) at 37 °C for 18 h. Digestion products were extracted using 1% formic acid, 2% acetonitrile followed by 1% formic acid and 50% acetonitrile. The recovered peptides were pooled, vacuum-centrifuged, dried and then resuspended into 12 µl of 0.1% formic acid, and 5 µl were analyzed by mass spectrometry. Peptide samples were separated by online reversed-phase (RP) nanoscale capillary liquid chromatography (nanoLC) and analyzed by electrospray mass spectrometry (ES MS/MS). The experiments were performed with an Ekspert NanoLC425 (Eksigent) coupled to a 5,600 + mass spectrometer (Sciex, Framingham, MA, USA) equipped with a nanoelectrospray ion source. Peptide separation took place on a self-packed picofrit column (New Objective) with reprosil 3u, 120A C18, 15 cm × 0.075 mm internal diameter, (Dr Maisch). Peptides were eluted with a linear gradient from 5–35% solvent B (acetonitrile, 0.1% formic acid) for 35 min at a flow rate of 300 nL/min. Mass spectra were acquired by a data dependent acquisition mode using Analyst software version 1.7. Each full scan mass spectrum (400 to 1,250 m/z) was followed by collision-induced dissociation of the twenty most intense ions. Dynamic exclusion was set for a period of 12 s and a mass tolerance of 100 ppm.

### Database searching

MGF peak list files were created using Protein Pilot version 4.5 software (Sciex). MGF sample files were then analyzed using Mascot (Matrix Science, London, UK; version 2.5.1). Mascot was searched with a fragment ion mass tolerance of 1.0 Da and a parent ion tolerance of 1.0 Da. Scaffold (version Scaffold_4.8.4, Proteome Software Inc., Portland, OR) was used to validate MS/MS based peptide and protein identifications based on the *Leishmania Infantum* TriTrypDB (version 9.0 released April 2016, 8,589 entries). Peptide identifications were accepted if they could be established at greater than 5.0% probability to achieve an FDR less than 1.0% by the Scaffold Local FDR algorithm. Protein identifications were accepted if they could be established at greater than 99.0% probability to achieve a FDR less than 1.0% and contained at least 2 identified peptides. Protein probabilities were assigned by the Protein Prophet algorithm^79^. Proteins that contained similar peptides and could not be differentiated based on MS/MS analysis alone were grouped to satisfy the principles of parsimony.

### 3D homology modeling and protein–protein docking

To construct 3D models of *L. infantum* JPCM5 proteins *Li*UFD1 (LinJ.36.6780), *Li*p47 (SEP domain, LinJ.22.0200), *Li*FAF2 (UBX domain, LinJ.35.1960), *Li*PUB1 domain (LinJ.09.1060), *Li*NPL4 (LinJ.25.1320) and *Li*VCP proteins, we searched for their orthologs in PDB database using BlastP and Delta-Blast. Then, 3D structure models were built using the modelling software MODELLER^80^ based on their homologous structure, PDBid : 5C1B_V, 1S3S_H, 2MX2_A, 2HPL_B, 6CDD_ A and 5C18_D, respectively. The QPTFAGAGRTL SHP1 peptide of *Li*UFD1 and the DDLYS peptide of *Li*VCP were modeled using as templates PDBid: 5C1B_V and 2HPL, respectively. The *Li*VCP hexamer was obtained by superposition with the *H. sapiens* p97 hexamer (PDBid: 5C18). The quality of the models was assessed by Ramachandran plot analysis through PROCHECK^81^. Proteins and peptides of *L. infantum* were docked into *Li*VCP using HDOCK^82^ and HPEPDOCK^83^, respectively. The electrostatic potential surfaces of 3D models and images were generated with PyMOL software (https://pymol.org/).

### Multiple alignments and phylogeny

We used the amino acid sequence of each *L. infantum* JPCM5 protein to search by BlastP and Delta-Blast for close homologs in bacteria, archaea, fungi, plants and animals. The sequences extracted from databases were aligned with Clustal Omega^84^. To establish the phylogenetic relationships between *L. infantum* proteins and those of prokaryotic and other eukaryotic organisms, the sequences were aligned with Clustal Omega^84^ and a phylogenetic tree was constructed using PhyML^85^. The gene ontology (GO) term enrichment analysis was performed using the tool provided by TriTrypDB (https://tritrypdb.org) with Fisher exact test filtering for false discovery rate (FDR) lower than 0.05. Cellular Component, Molecular Function and Biological Process were carried out separately.

### Experimental design and statistical rationale

*Filter for LiVCP IP-MS/MS:* Seven IP-MS/MS studies were considered for *Li*VCP (Supplementary Table S2). To select proteins that specifically associate with *Li*VCP, we calculated the average number of peptides for each protein in all seven experiments, and then excluded all proteins also found in five independent IP-MS/MS studies conducted with HA-tagged proteins unrelated to the VCP complex. From the selected 24 *Li*VCP specific interacting proteins, 15 with an average of exclusive unique peptide count (EUPC) ≥ 2.0 were classified as the *Li*VCP core and are presented in Fig. 1C and Supplementary Table S2.

*Filter for LiVCP cofactors IP-MS/MS:* Two independent experiments were performed for each *Li*VCP cofactor (*Li*p47, *Li*FAF2, *Li*UFD1 and *Li*PUB1) and only proteins with an average of exclusive unique peptide count EUPC ≥ 2.0 in both experiments for each cofactor were considered (Supplementary Table S2). To exclude non-specific partners, IP-MS/MS studies with proteins unrelated to *Li*VCP complexes were carried out and proteins with an average of EUPC > 3.6 (*Li*VCP threshold) were used to filter out non-specific *Li*VCP cofactor associated proteins. The remaining proteins co-immunoprecipitated with *Li*p47, or *Li*FAF2, or *Li*UFD1 and or *Li*PUB1 were considered as putative *Li*VCP cofactors. Cytoscape v 3.5.1^86^ was used to plot network analyses of all new interactions for *Li*VCP, *Li*p47, *Li*FAF2, *Li*UFD1 and *Li*PUB1.

*Filter for network plotting*: To decrease the number of false positive results in the *Li*VCP network building, we only show proteins that were not co-immunoprecipitated with all unrelated HA-tagged proteins (average of EUPC = 0.0). From the total number of proteins co-immunoprecipitated with *Li*p47, or *Li*UFD1 or *Li*FAF2 and or *Li*PUB1, only 10, 17, 23 and 9, respectively were considered together with the 24 *Li*VCP specific interactors (Fig. 1, Table 1, and Supplementary Table S2) to build the topology of *Li*VCP network. No statistical analysis was needed.

## Supplementary information

Supplementary file1

Supplementary file2

Supplementary file3

Supplementary file4

Supplementary file5

Supplementary file6

## Data Availability

The mass spectrometry proteomics data have been deposited to the ProteomeXchange Consortium via the PRIDE^87^ partner repository with the dataset identifier PXD013731. The Reviewer account details are Username: reviewer11708@ebi.ac.uk, Password: Zm5uR3hc.

## Abbreviations

AAA: ATPases Associated with diverse cellular Activities
ER: Endoplasmic reticulum
ERAD: ER-associated degradation
EUPC: Exclusive unique peptide count
FAF: Fas-associated factor
FCS: Fetal calf serum
IP: Immunoprecipitation
*Li*VCP: *Leishmania infantum* VCP
MPN: Mpr1, Pad1 N-terminal domain
NPL4: Nuclear protein localization protein 4
OMM: Outer mitochondrial membrane
OTU1: Ovarian tumor domain-containing protein 1
PP1: Protein phosphatase type 1
PUB: PNGase/UBA or UBX-containing proteins
PUL: PLAP, Ufd3p, and Lub1p
SEP: Shp, eyes-closed, p47
SHP: BS1, binding segment 1
SPFH: Stomatin, prohibitin, flotillin, HflC/K
UBA: Ubiquitin-associated
UBL: Ubiquitin-like domain
UBX: Ubiquitin regulatory X
UBXD: Ubiquitin X domain
UBXL: Ubiquitin regulatory X-like
UFD1: Ubiquitin fusion degradation protein 1
UPS: Ubiquitin Proteasome System
VBM: VCP-binding motif
VCP: Valosin-containing protein
VIM: VCP-interacting motif
