# Supplementary material for: The AAA + ATPase valosin-containing protein (VCP)/p97/Cdc48 interaction network in Leishmania
Source: Sci Rep. 2020 Aug 4;10:13135. doi: 10.1038/s41598-020-70010-4 (PMC7403338; doi:10.1038/s41598-020-70010-4)
Supplement: Supplementary file 1 — Supplementary file1 [file 41598_2020_70010_MOESM1_ESM.pdf]

Supplementary Data

The AAA+ ATPase Valosin-containing protein (VCP)/p97/Cdc48 interaction network in *Leishmania*

Bruno Guedes A. Aguiar[1,2,3], Carole Dumas[1,2], Halim Maaroufi[4], Prasad K. Padmanabhan[1,2] and Barbara Papadopoulou[1,2]*

[1]Division of Infectious Disease and Immunity, CHU de Quebec Research Center-Laval University

[2]Department of Microbiology-Infectious Disease and Immunology, Faculty of Medicine, University Laval, Quebec, QC. Canada G1V 4G2

[3]Department of Community Medicine, Federal University of Piauí, Teresina, Brazil

[4]Institut de Biologie Intégrative et des Systèmes (IBIS), Laval University, Quebec, QC. Canada

List of the Supplementary material included:

Supplementary Tables

Supplementary Table S1. Overlap between the BioGRID VCP interactors and *Leishmania* homologs. This spreadsheet presents the analysis of the overlap between interactors reported for the *H. sapiens* VCP from the Biogrid interaction dataset 3.4 and their similarities with *Leishmania* homologous proteins.

Supplementary Table S2. Proteins potentially interacting with the *Leishmania Li*VCP and its main cofactors as determined by immunoprecipitation and LC-MS/MS studies. This spreadsheet contains data from all IP-MS/MS experiments reported in this study for *Li*VCP, its cofactors and unrelated proteins used as filters.

Supplementary Table S3. GO-term analysis (cellular component, molecular function and biological process) of the *Li*p47, *Li*FAF2, *Li*UFD1 and *Li*PUB1 proteomes using the tool provided by TriTrypDB. Only proteins co-immunoprecipitated either with *Li*p47 or *Li*FAF2 or *Li*UFD1 and or *Li*PUB1 but not found in the IPs of the unrelated control proteins as described in Figure 3A were considered for these analyses. Raw data are presented.

Supplementary Table S4. Ramachandran plot analysis of all the modeled proteins. The quality of the 3D models for *Li*VCP and its main cofactors was assessed by Ramachandran plot analysis through the PROCHECK software module.

Supplementary Table S5. Proteins used for constructing the *Li*VCP interaction network classified based on their intersection. This spreadsheet contains the list of proteins used for the *Li*VCP network construction considering the presence/absence of different proteins on our multiple co-IP-MS/MS experiments.

Supplementary Table S6. Primers used in this study.

Supplementary Table S4. Ramachandran plot analysis of all the modeled proteins.

**Modeled proteins**	**Residues in most favoured regions (%)**	**Residues in additional allowed regions (%)**	**Residues in generously allowed regions (%)**	**Residues in disallowed regions (%)**
*Li*VCP	94.3	4.8	0.6	0.3
*Li*PUB1	88.9	9.9	1.2	0.0
*Li*p47	87.0	8.7	4.3	0.0
*Li*FAF2	90.0	10.0	0.0	0.0
*Li*UFD1	87.1	11.2	1.2	0.6
*Li*NPL4	84.1	15.1	0.5	0.3

The quality of the models was assessed by Ramachandran plot analysis through the PROCHECK software module. The results of the Ramachandran plot showed that 95.7% to 100% of the amino acid residues of the constructed 3D homology models using alignments in Supplementary Figures S8, S10, S12, S14 and S17 are found in most favoured and additional allowed regions. These results indicate that the predicted structures are reliable. Moreover, Ramachandran plot also showed that in our 3D homology models the amino acid residues implicated in interactions are in most favoured regions.

Supplementary figures:

Supplementary Figure S1. Known interacting partners of the *H. sapiens* VCP/p97 identified as putative cofactors of the *L. infantum Li*VCP protein.

Supplementary Figure S2. Ectopic expression of HA-tagged *Li*UFD1, *Li*p47, *Li*FAF2, *Li*NPL4 and *Li*PUB1 cofactors of the *Leishmania Li*VCP.

Supplementary Figure S3. Phylogenetic analysis of p47 homologs from different eukaryotes.

Supplementary Figure S4. Phylogenetic relationships between FAF2 homologs from different eukaryotes.
Supplementary Figure S5. Phylogenetic relationships between UFD1 homologs from different eukaryotes.

Supplementary Figure S6. Phylogenetic relationships between different NPL4 eukaryotic homologs.

Supplementary Figure S7. Phylogenetic relationships between PUB homologs from different eukaryotes.

Supplementary Figure S8. Sequence alignment of the *L. infantum Li*p47 with homologs from other eukaryotes.

Supplementary Figure S9. 3D homology modeling and docking predictions for *Li*p47 into *Li*VCP.

Supplementary Figure S10. Sequence alignment of the *L. infantum Li*FAF2 with homologs from other eukaryotes.

Supplementary Figure S11. 3D homology modeling and docking predictions for *Li*FAF2 into *Li*VCP.

Supplementary Figure S12. Sequence alignment of the *L. infantum Li*UFD1 with homologs from other eukaryotes.

Supplementary Figure S13. 3D homology modeling and docking predictions for *Li*UFD1 into *Li*VCP.

Supplementary Figure S14. Sequence alignment of the *L. infantum Li*NPL4 with homologs from other eukaryotes.

Supplementary Figure S15. 3D homology modeling and docking predictions for *Li*NPL4 into *Li*VCP.

Supplementary Figure S16. The *Leishmania* VCP cofactors *L*iUFD1 and *Li*NPL4 associate with poly-ubuquitinated chains.

Supplementary Figure S17. Sequence alignment of the *L. infantum Li*PUB1 protein with homologs from other eukaryotes.

Supplementary Figure S18. 3D homology modeling and docking predictions for *Li*PUB1 into *Li*VCP.

Supplementary Figure S19. Gene Ontology (GO) analysis.

Supplementary Figure S20. Uncropped blots.

Supplementary Figures

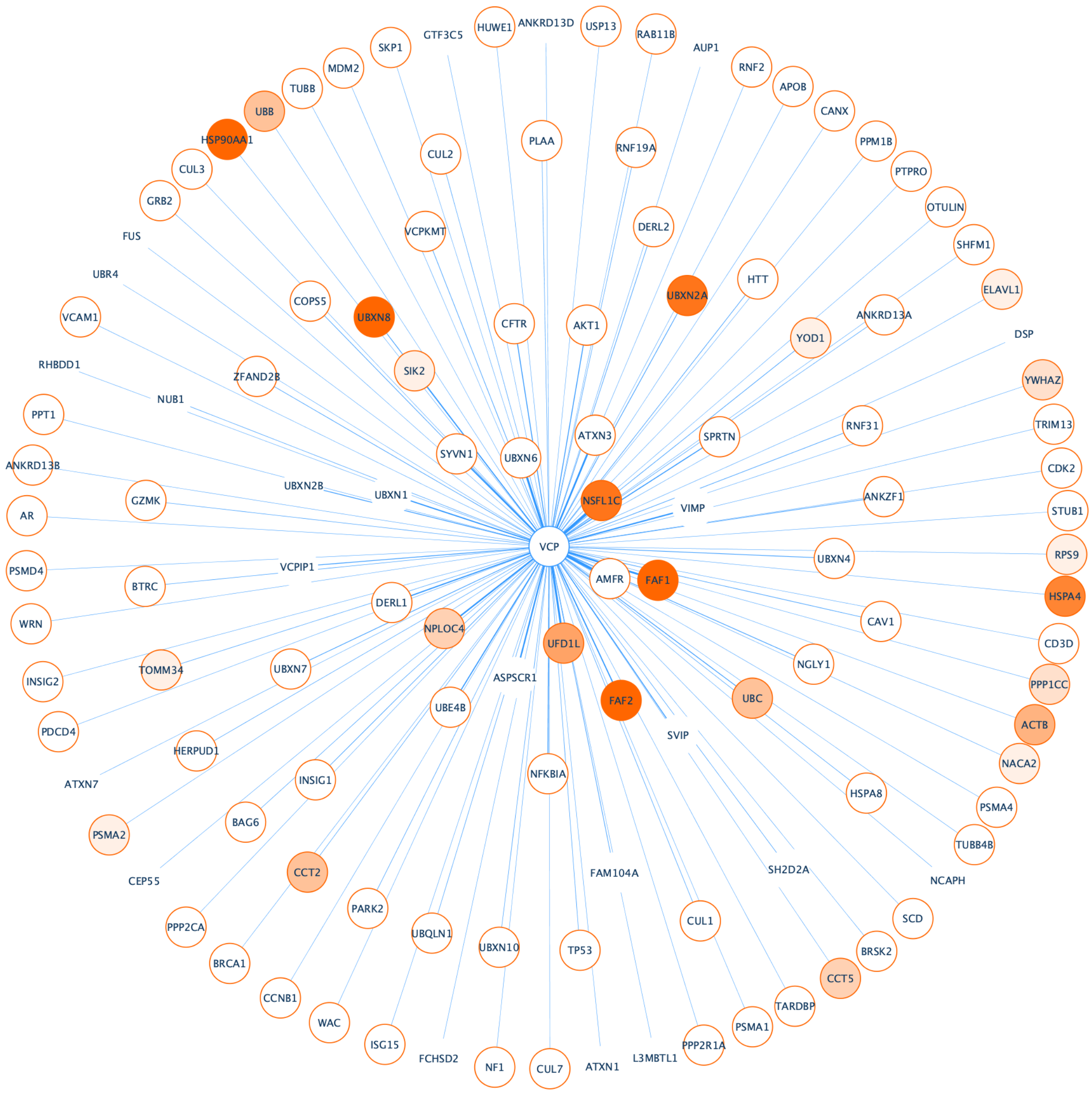

Supplementary Figure S1. Known interacting partners of the *H. sapiens* VCP/p97 identified as putative cofactors of the *L. infantum Li*VCP protein. Network analyses of the known physical (1091) and genetic (2) interactions of the human VCP ortholog from the Biogrid interaction dataset 3.4 (thebiogrid.org) plotted on Cytoscape version 3.5.1 (Shannon *et al.,* 2003). Edge length and thickness represents interaction with VCP/p97. The thicker the edge the more experimental evidence has been reported on Biogrid and closer they were placed to the center. Orange circles represent homology with any protein of the *Leishmania infantum* genome (see Supplementary Table S1). Orange filled circles represent *Li*VCP interactions reported in this work. Darker shades of orange represent higher average of peptides identified by mass spectrometry in our *Li*VCP-HA co-immunoprecipitates (see Supplementary Table S2 for more details). 0-15: Average of exclusive unique peptides identified in *Li*VCP-HA immunoprecipitates followed by mass spectrometry.

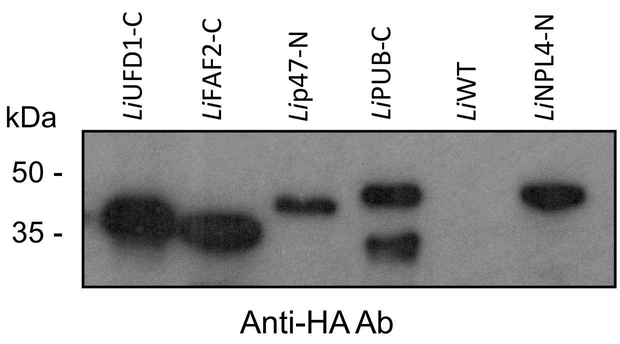

Supplementary Figure S2. Ectopic expression of HA-tagged *Li*UFD1, *Li*p47, *Li*FAF2, *Li*NPL4 and *Li*PUB1 cofactors of the *Leishmania Li*VCP. Western blotting with an anti-HA antibody to detect C- or N- terminally HA-tagged *Li*UFD1, *Li*p47, *Li*FAF2, *Li*PUB1 and *Li*NPL4 cofactor proteins cloned in pSPαZEOα vector and transfected into *L. infantum* promastigotes. The *L. infantum* strains expressing these vectors were used for co-IP/LC-MS/MS analysis. Shown here are the most representative results from 3-5 similar experiments.

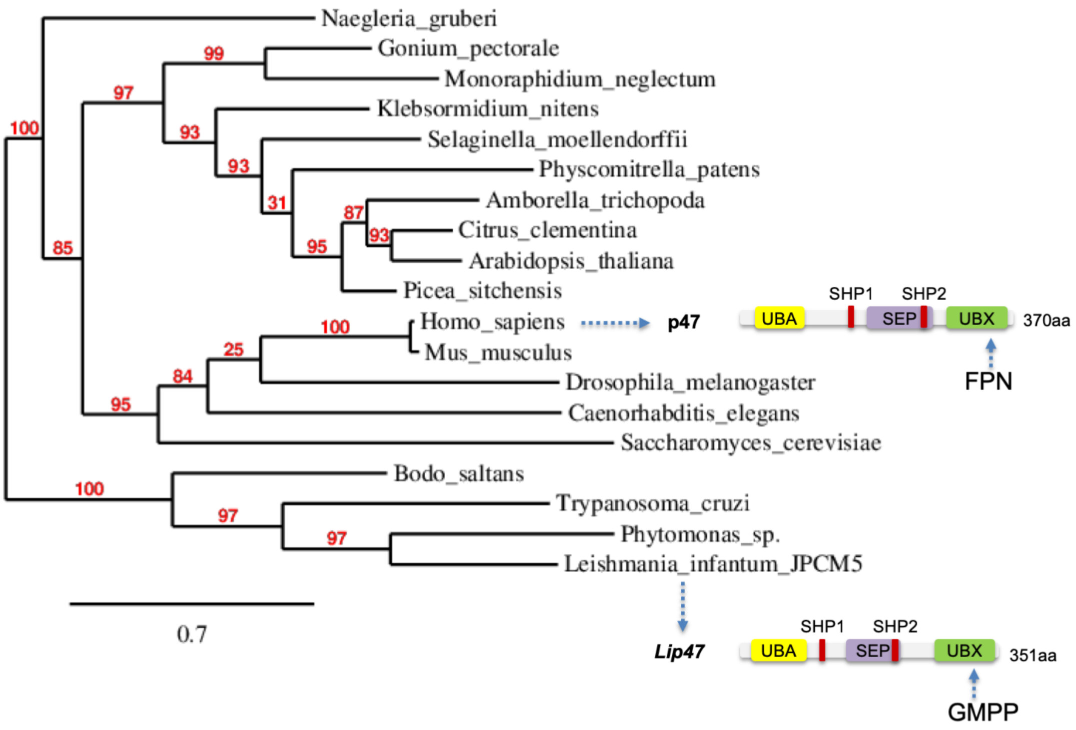

Supplementary Figure S3. Phylogenetic analysis of p47 homologs from different eukaryotes. Sequence alignment of Supplementary Figure S8 was used to construct a maximum likelihood phylogenetic tree. The UBX binding site FPN in the human p47 protein and the UBX binding MPN (Mpr1/Pad1 N-terminal) domain in the *Leishmania Li*p47 protein are indicated by arrow. The analysis used a WAG substitution model and the statistical confidence of the nodes was calculated using the aLRT test.

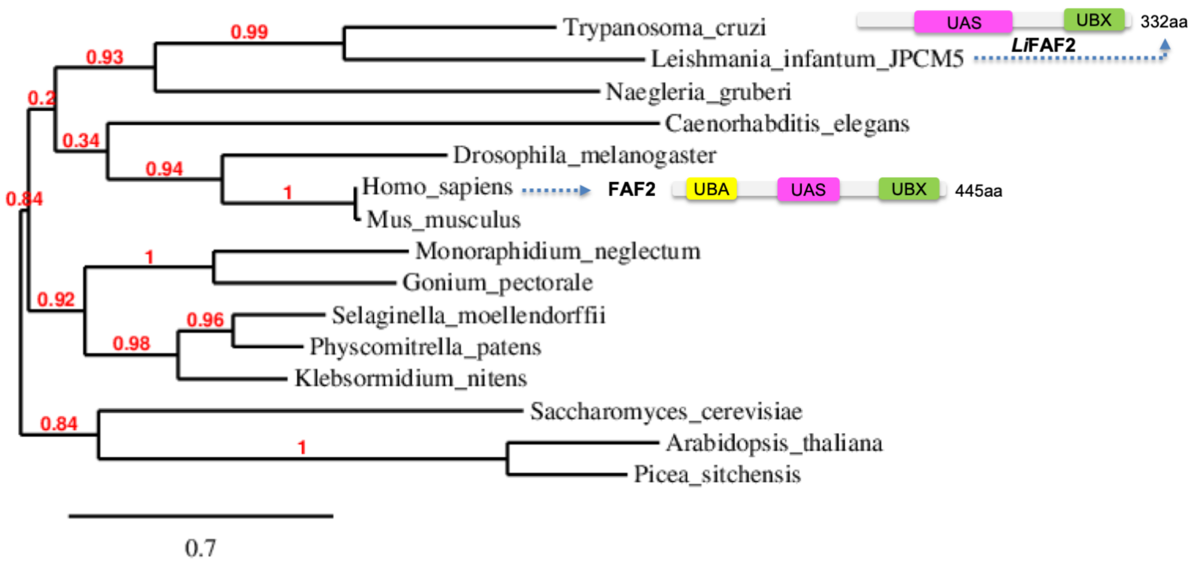

Supplementary Figure S4. Phylogenetic relationships between FAF2 homologs from different eukaryotes. Sequence alignment of Supplementary Figure S10 was used to construct a maximum likelihood phylogenetic tree. The *Leishmania Li*FAF2 protein lacks the ubiquitin associated (UBA) domain. The analysis used a WAG substitution model, and the statistical confidence of the nodes was calculated using the aLRT test.

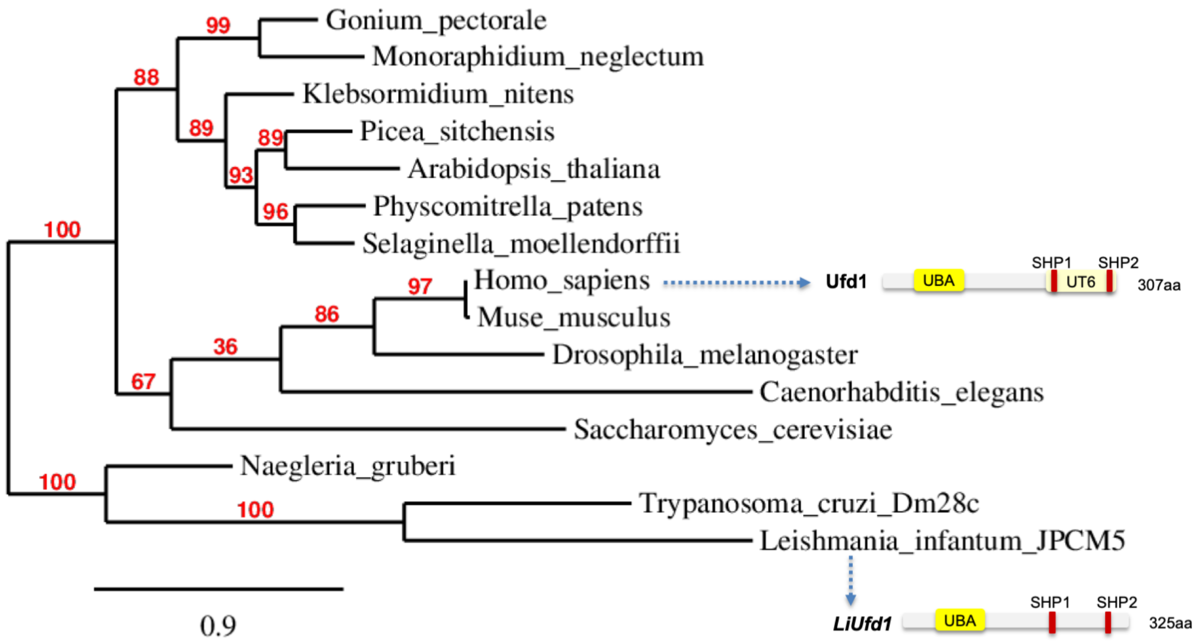

Supplementary Figure S5. Phylogenetic relationships between UFD1 homologs from different eukaryotes. Sequence alignment of Supplementary Figure S12 was used to construct a maximum likelihood phylogenetic tree. The analysis used a WAG substitution model, and the statistical confidence of the nodes was calculated using the aLRT test.

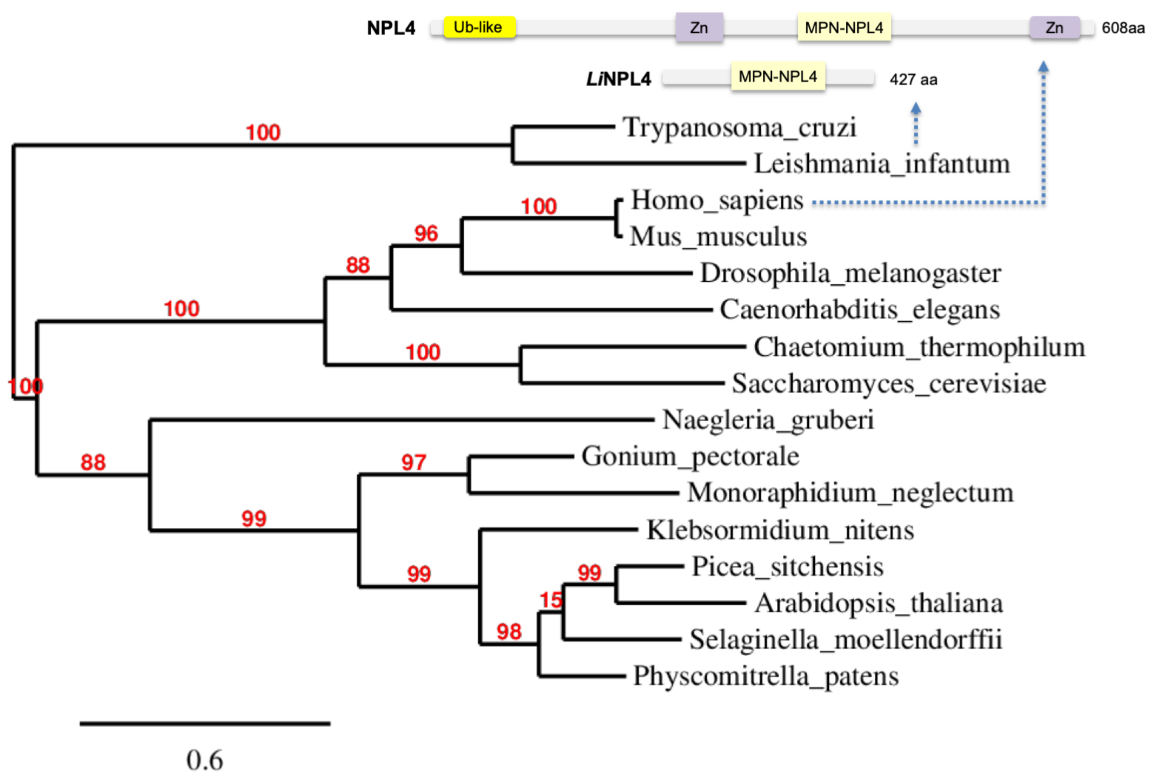

Supplementary Figure S6. Phylogenetic relationships between different NPL4 eukaryotic homologs. Sequence alignment of Supplementary Figure S14 was used to construct a maximum likelihood phylogenetic tree. The *Leishmania Li*NPL4 homolog lacks the ubiquitin regulatory domain (UBX)-like and the two zinc finger motifs. The analysis used a WAG substitution model, and the statistical confidence of the nodes was calculated using the aLRT test.

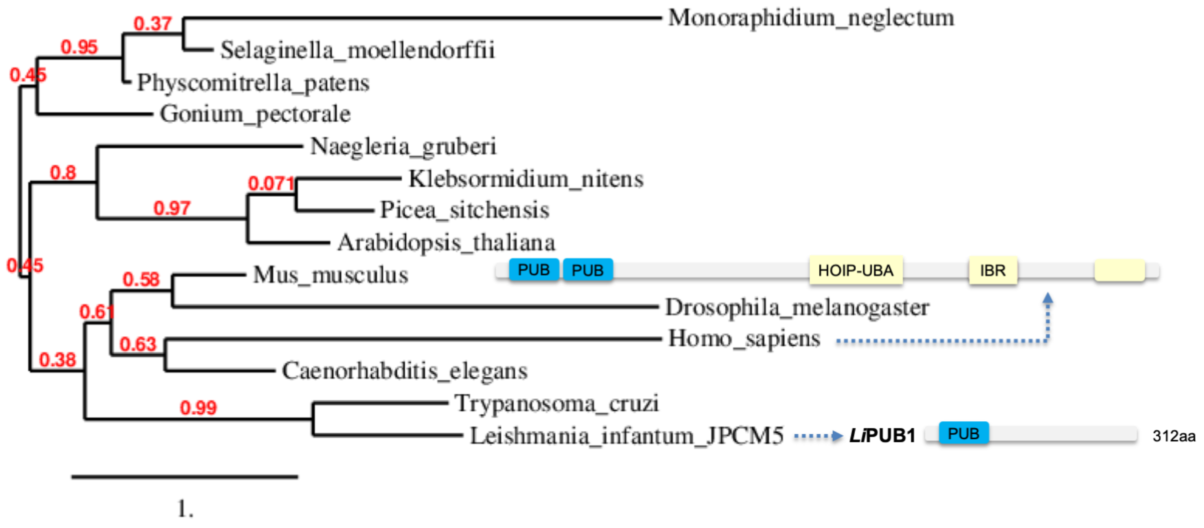

Supplementary Figure S7. Phylogenetic relationships between PUB homologs from different eukaryotes. Sequence alignment of Supplementary Figure S17 was used to construct a maximum likelihood phylogenetic tree. The *Leishmania*, *Li*PUB1, protein which has no homolog in human. The analysis used a WAG substitution model, and the statistical confidence of the nodes was calculated using the aLRT test.

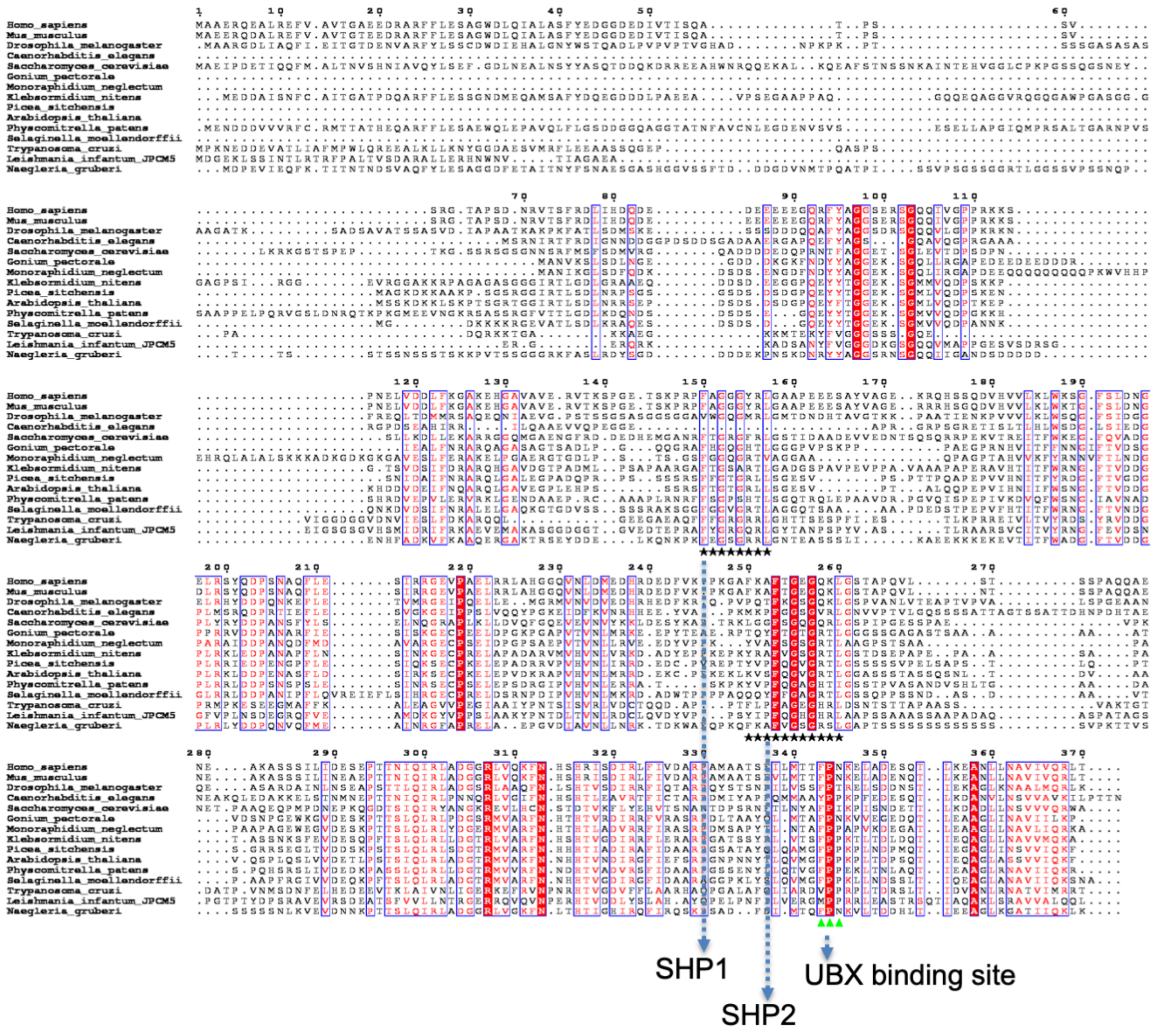

Supplementary Figure S8. Sequence alignment of the *L. infantum Li*p47 with homologs from other eukaryotes. Sequence alignment was done using Clustal Omega. Highly conserved amino acid residues are shown in red and boxed in blue. Black asterisks represent residues of SHP1 and SHP2 motifs and green triangles indicate the UBX binding site. The figure was prepared with ESPript (http://espript.ibcp.fr).

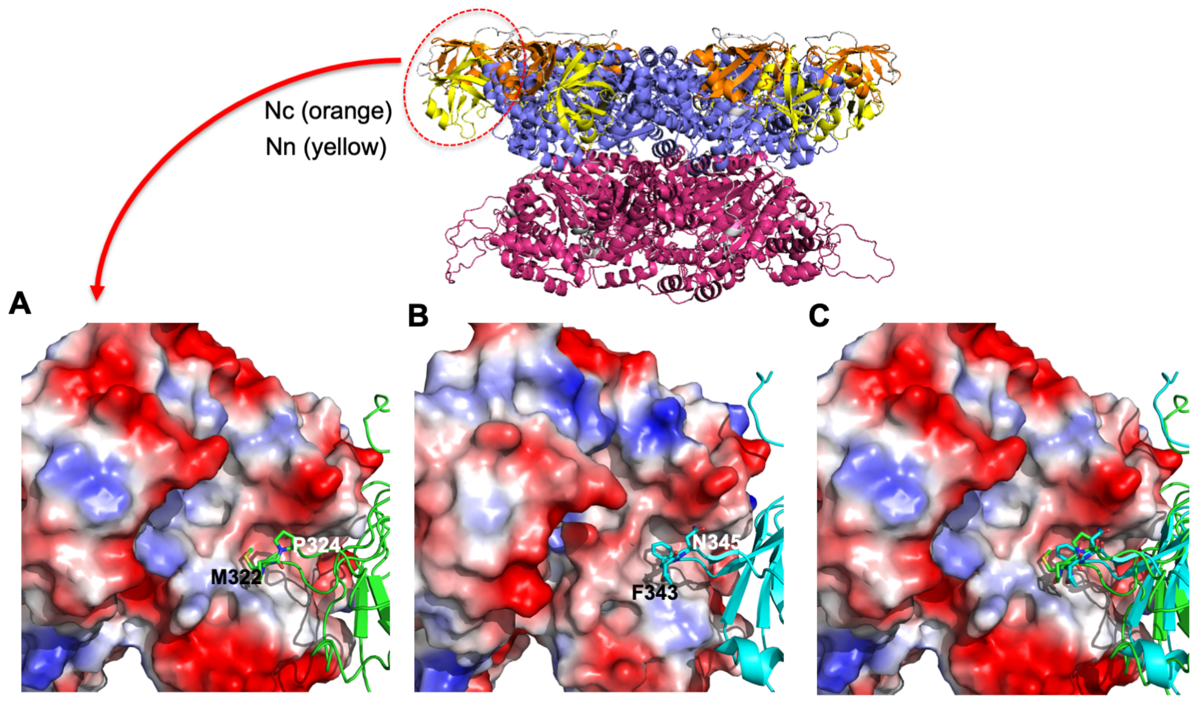

Supplementary Figure S9. 3D homology modeling and docking predictions for *Li*p47 into *Li*VCP. (**A**) 3D model of interactions between the N-terminus of *Li*VCP (electrostatic potential) and the GMPP motif of *Li*p47 UBX domain (in green). (**B**) PDBid 1S3S structure representing the interactions between the N-terminus of VCP and the FPN motif within the UBX binding site of p47 (in cyan). (**C**) Superposition of structures represented in (A) and (B).

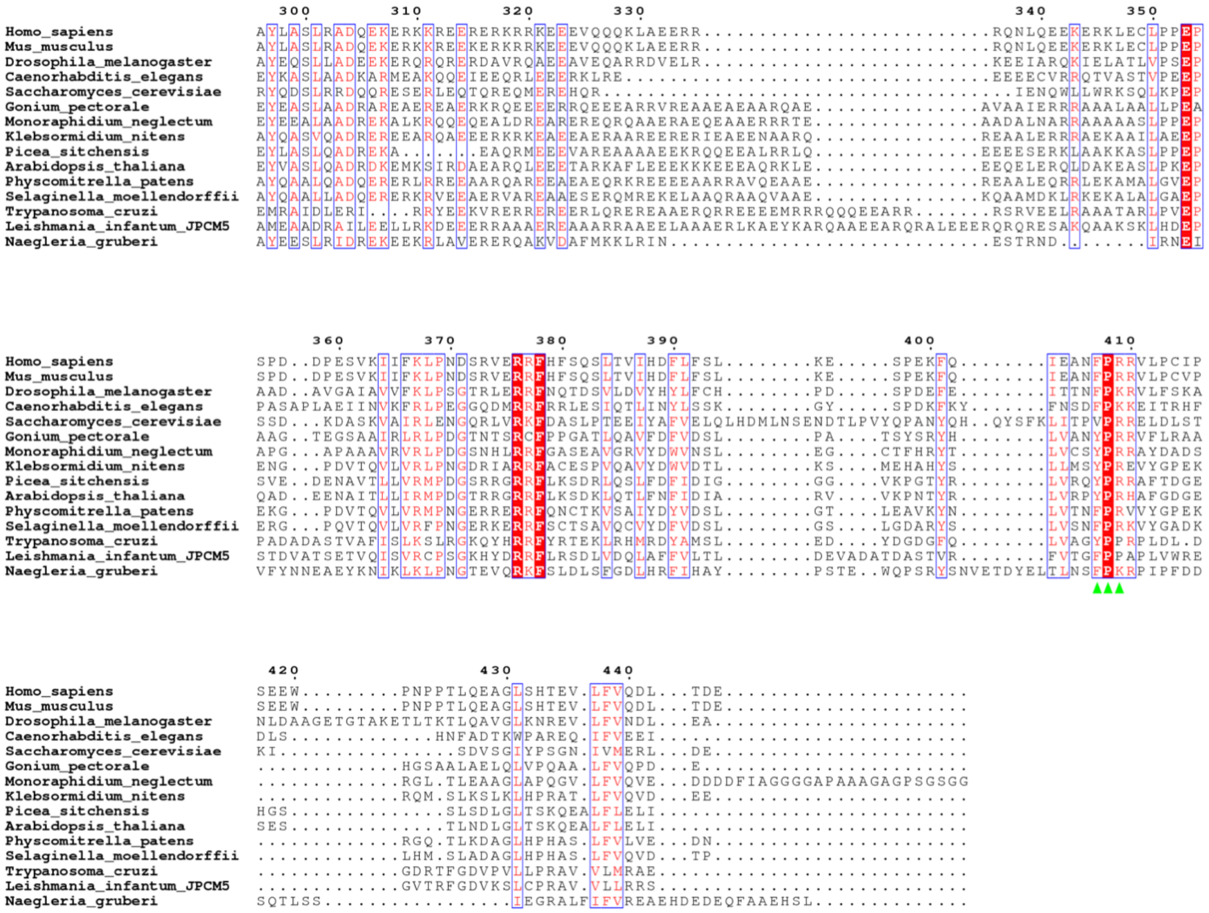

Supplementary Figure S10. Sequence alignment of the *L. infantum Li*FAF2 with homologs from other eukaryotes. Sequence alignment was done using Clustal Omega. Highly conserved amino acid residues are shown in red and boxed in blue. Green triangles represent the UBX binding site FPP in *L. infantum*. The figure was prepared with ESPript (http://espript.ibcp.fr). Here, only the conserved region from aa 300 to 440 is shown, the rest of the protein sequence being more divergent between the different eukaryotic FAF proteins.

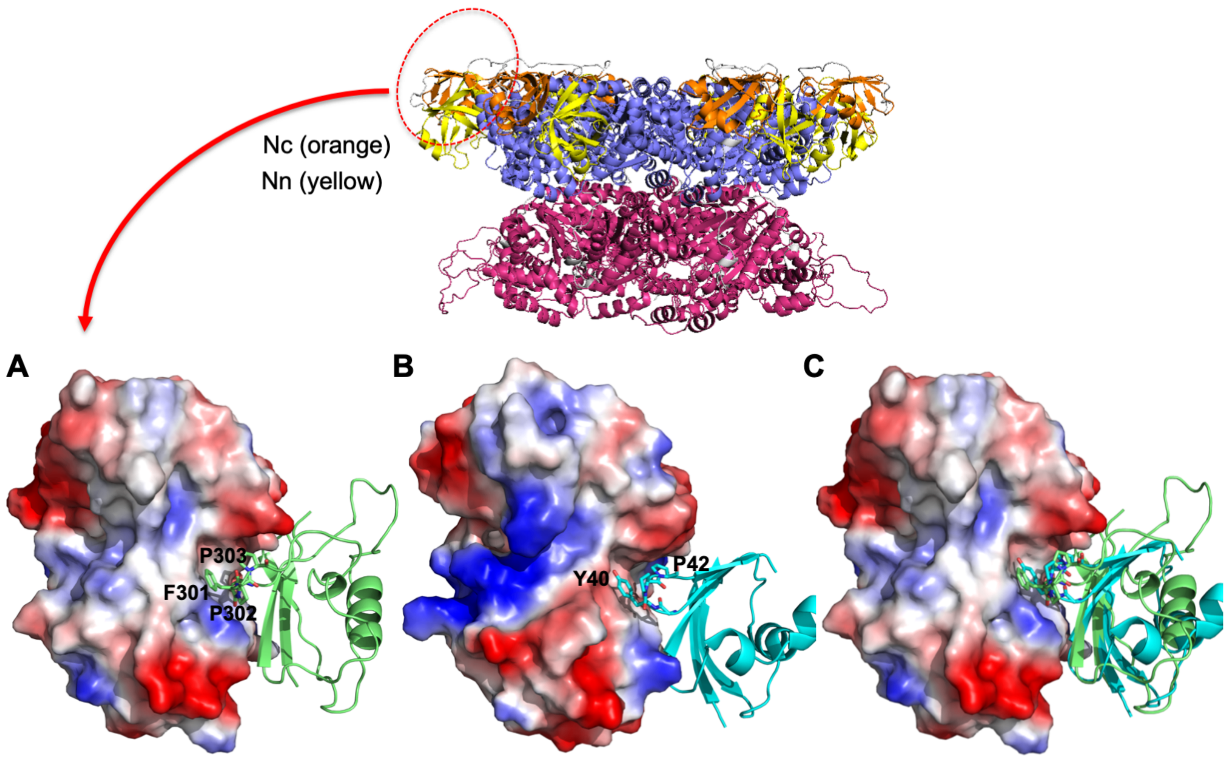

Supplementary Figure S11. 3D homology modeling and docking predictions for *Li*FAF2 into *Li*VCP. (**A**) 3D model of interactions between the N-terminus of *Li*VCP (electrostatic potential) and the GFPP motif of the UBX *Li*FAF2 domain (in green). (**B**) PDBid 4KDL structure representing the interactions between the N-terminus of VCP and the ^{40}GYPP^{43} motif of ovarian tumor domain-containing protein 1 (OTU1) (in cyan). (**C**) Superposition of structures represented in (A) and (B). For details, see www.rcsb.org/structure/4KDL.

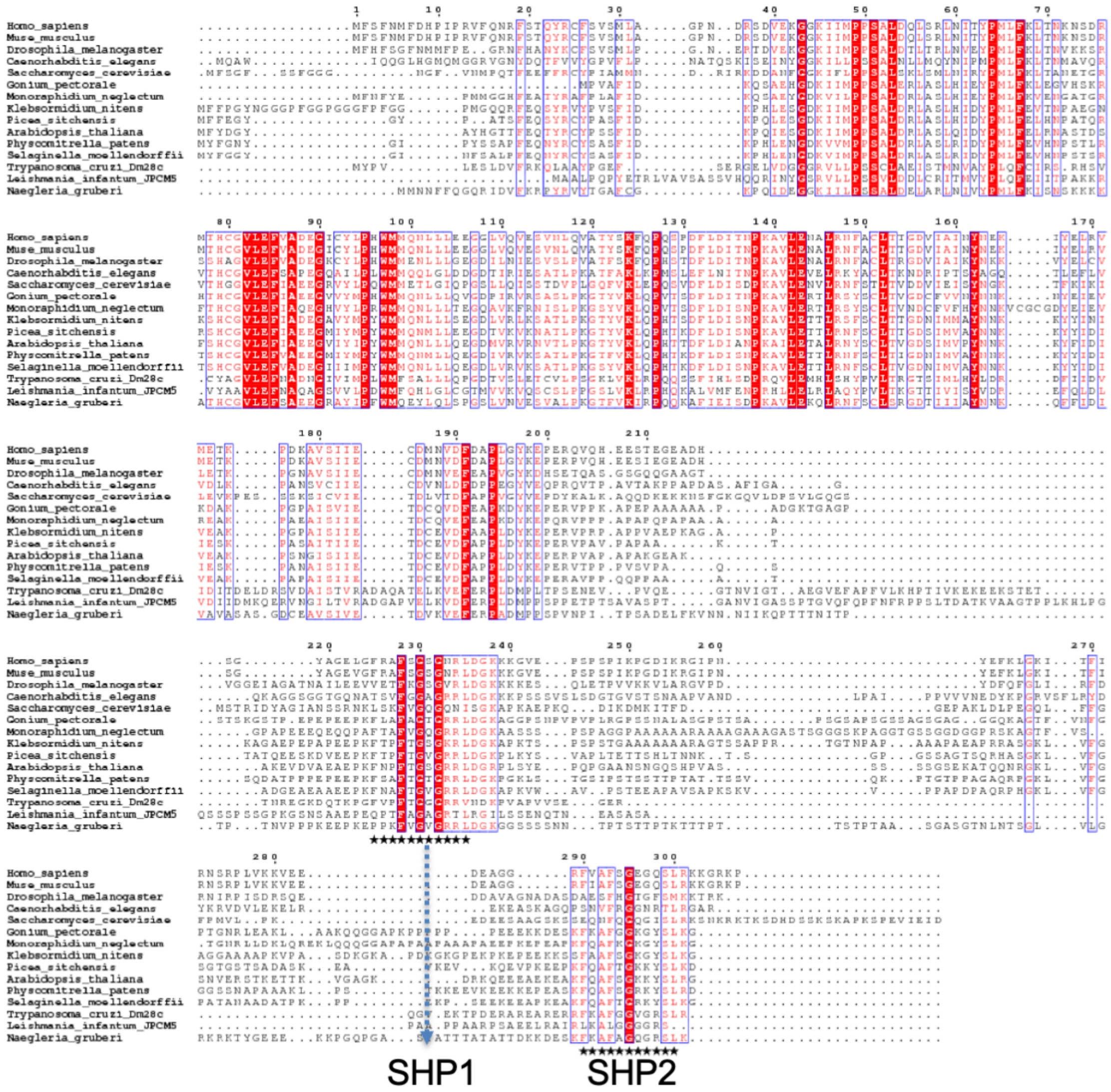

Supplementary Figure S12. Sequence alignment of the *L. infantum Li*UFD1 with homologs from other eukaryotes. Sequence alignment was done using Clustal Omega. Highly conserved amino acid residues are shown in red and boxed in blue. Black asterisks represent residues of SHP1 and SHP2 motifs. The figure was prepared with ESPript (http://espript.ibcp.fr).

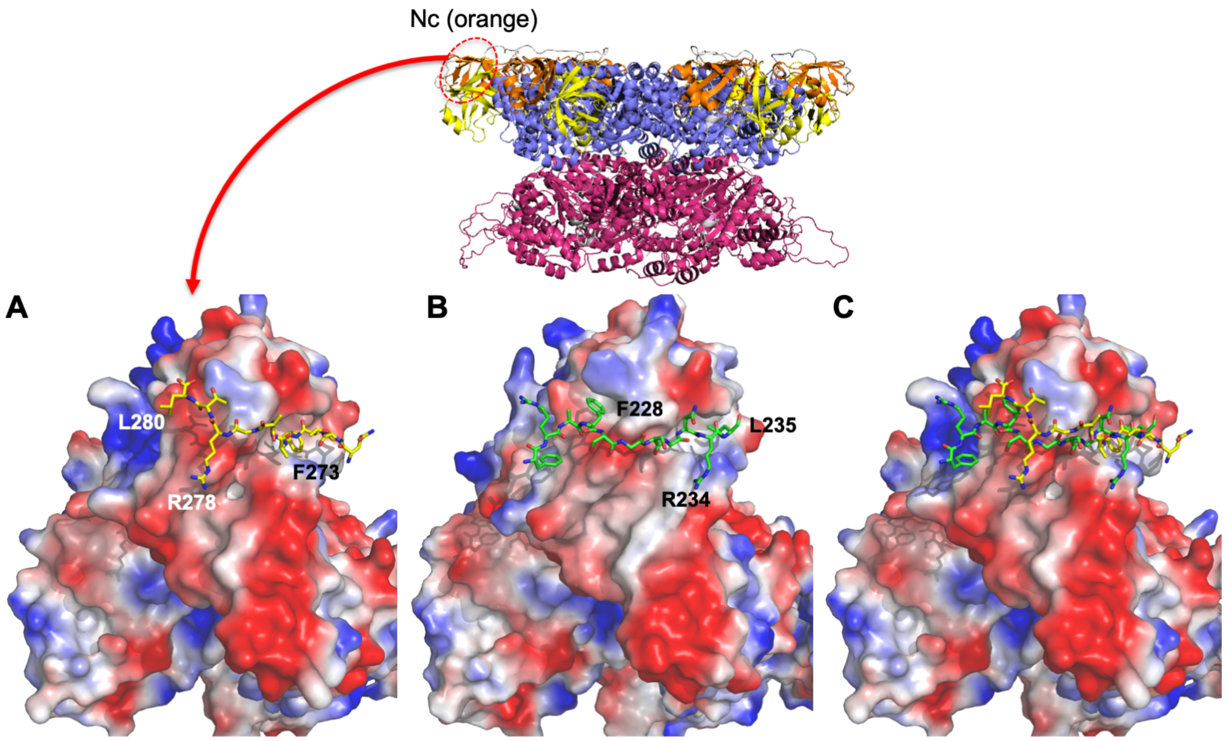

Supplementary Figure S13. 3D homology modeling and docking predictions for *Li*UFD1 into *Li*VCP. (**A**) 3D model of interactions between the N-terminus of *Li*VCP (electrostatic potential) and the QPTFAGAGRTL SHP1 motif of *Li*UFD1 (in yellow). (**B**) PDBid 5C1B structure representing the interactions between the N-terminus of VCP and the SHP domain of UFD1 (in green). (**C**) Superposition of structures represented in (A) and (B).

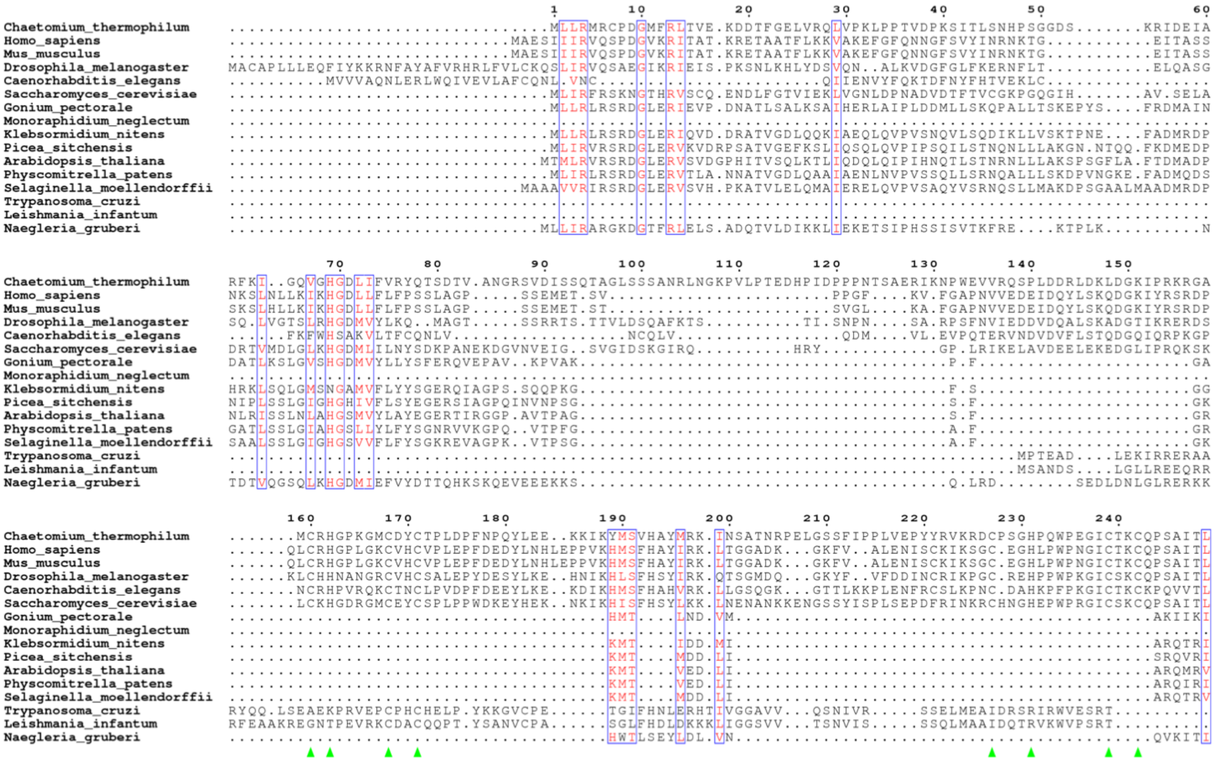

Supplementary Figure S14 1/3

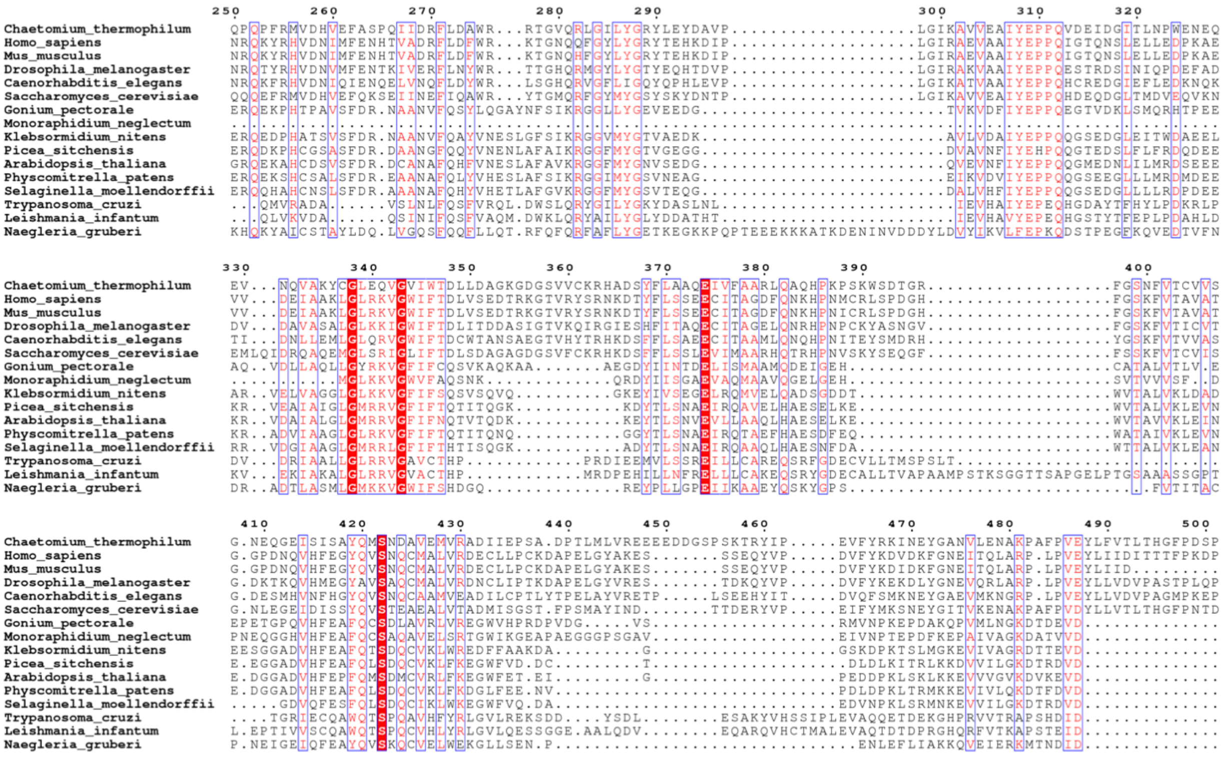

Supplementary Figure S14 2/3

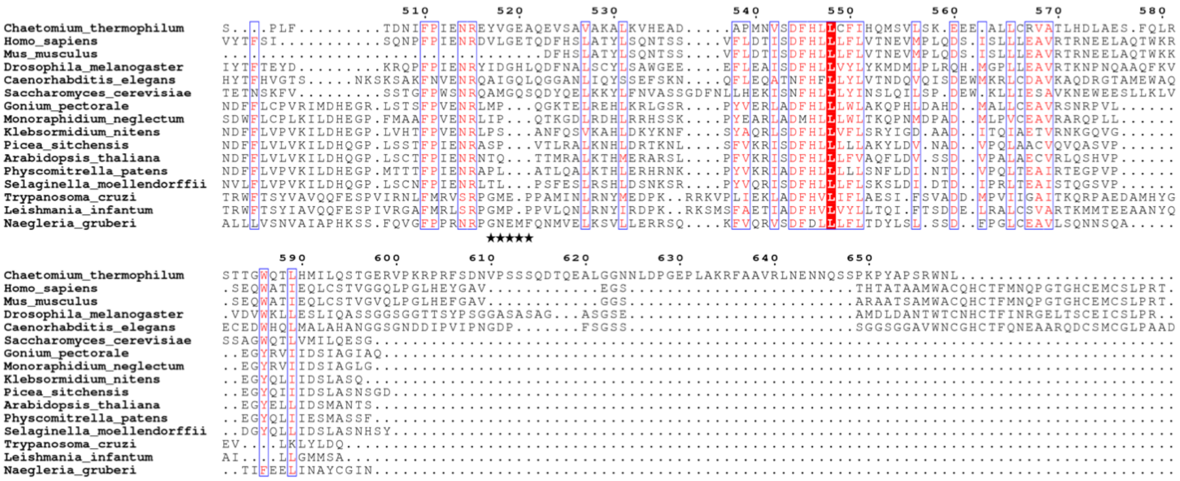

Supplementary Figure S14 3/3

Supplementary Figure S14. Sequence alignment of the *L. infantum Li*NPL4 with homologs from other eukaryotes. Sequence alignment was done using Clustal Omega. Highly conserved amino acid residues are shown in red and boxed in blue. Green triangles indicate the UBX binding site in other eukaryotic orthologs (the *Leishmania* NPL4 homolog lacks the UBX-like domain). Black asterisks represent the GMPP motif within the C-terminus of *Li*NPL4. The figure was prepared with ESPript (http://espript.ibcp.fr).

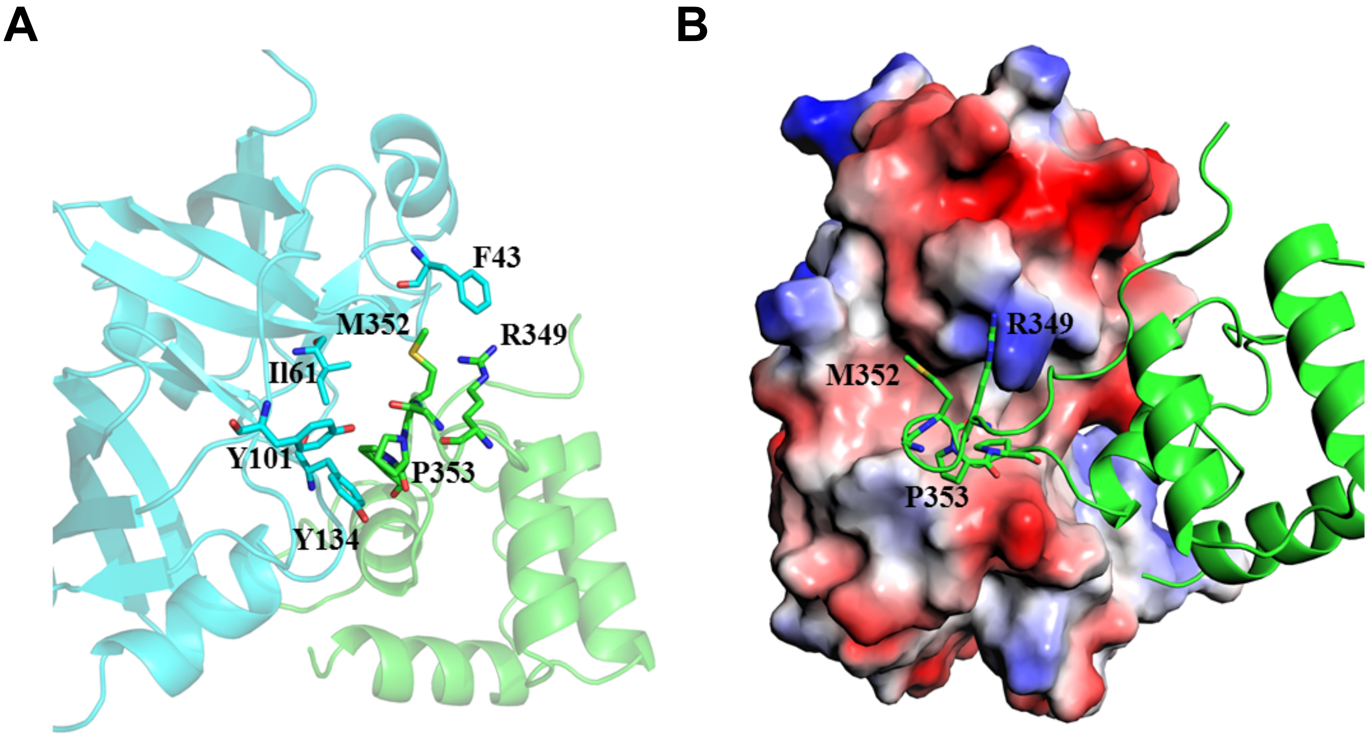

Supplementary Figure S15. 3D homology modeling and docking predictions for *Li*NPL4 into *Li*VCP. (**A**) Cartoon view of the interactions between the N-terminus of *Li*VCP (cyan) and the GMPP motif located at the C-terminus of *Li*NPL4 (green). (**B**) 3D model with the electrostatic potential surface representation of the *Li*VCP N-terminus. Negative and positive charges are shown in red and blue, respectively.

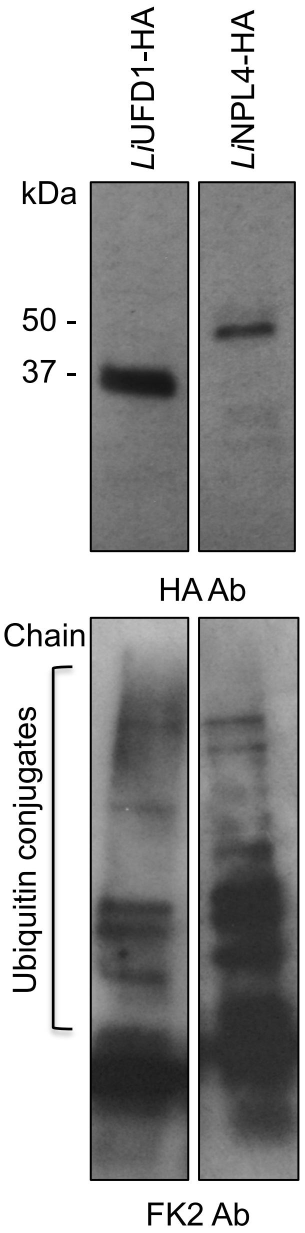

Supplementary Figure S16. The *Leishmania* VCP cofactors *Li*UFD1 and *Li*NPL4 associate with poly-ubiquitinated chains. Immunoprecipitation from *L. infantum* parasites expressing *Li*UFD1-HA or *Li*NPL4-HA followed by western blotting using an anti-HA antibody (top panel). Western blotting of the *Li*UFD1-HA or *Li*NPL4-HA immunoprecipitates using the FK2 antibody recognizing K^{29}-, K^{48}-, and K^{63}-linked mono- and poly-ubiquitin chains.

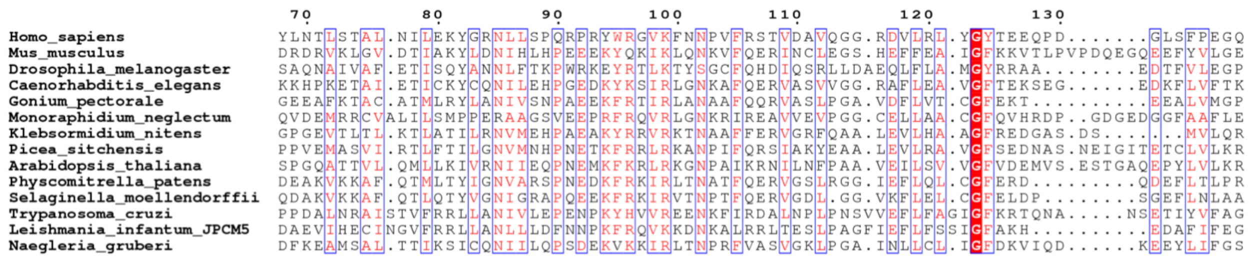

Supplementary Figure S17. Sequence alignment of the *L. infantum Li*PUB1 protein with homologs from other eukaryotes. Sequence alignment was done using Clustal Omega. Highly conserved amino acid residues are shown in red and boxed in blue. The figure was prepared with ESPript (http://espript.ibcp.fr). Here, only the conserved region from PUB domain is shown, the rest of the protein sequence being more divergent between different eukaryotic organisms.

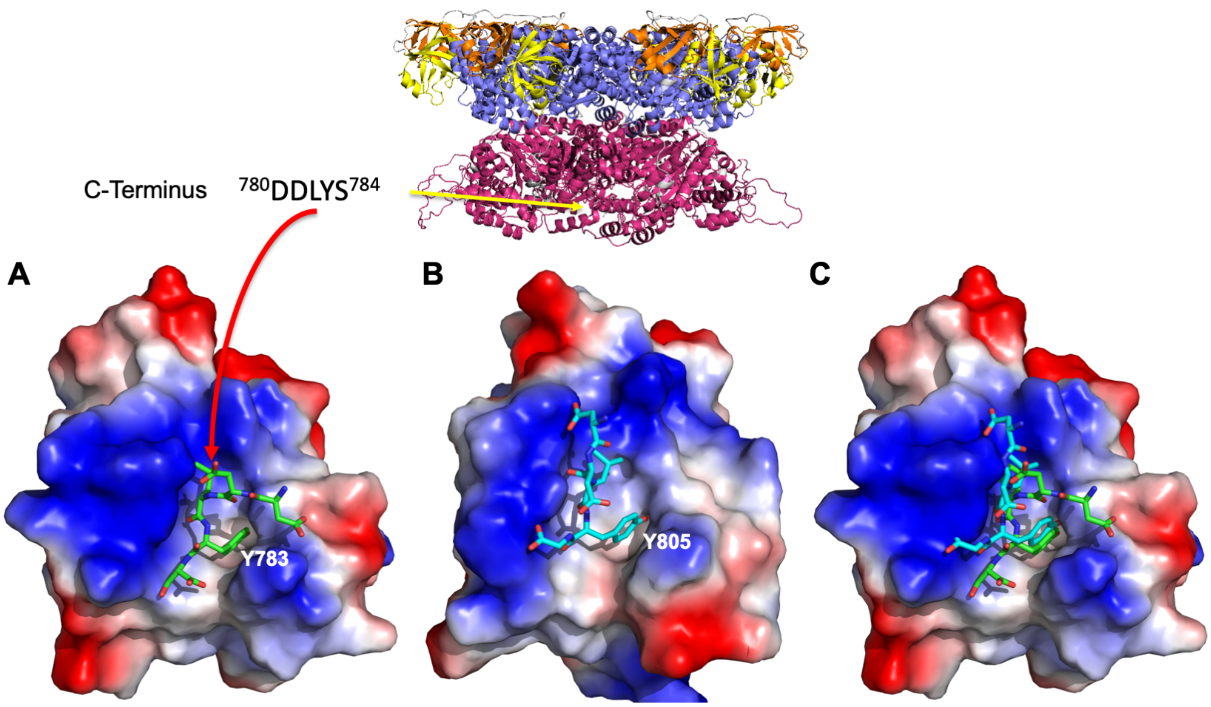

Supplementary Figure S18. 3D homology modeling and docking predictions for *Li*PUB1 into *Li*VCP. (**A**) 3D model of interactions between the *Li*PUB1 protein (electrostatic potential) and the C-terminal DDLYS motif of *Li*VCP (in green). (**B**) PDBid 2HPL structure representing the interactions between the PUB domain (electrostatic potential) and the C-terminal DDLYG motif of VCP (in cyan). (**C**) Superposition of structures represented in (A) and (B).

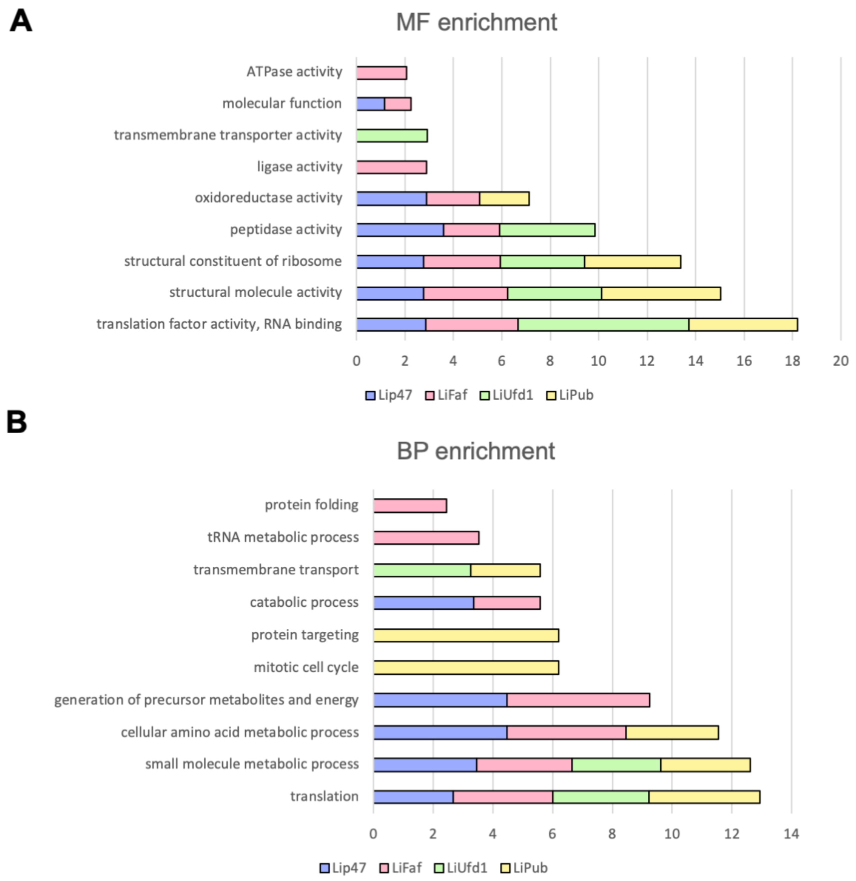

Supplementary Figure S19. Gene Ontology (GO) analysis. Biological process (A) and molecular function (B) analyses for proteins identified by mass spectrometry in *Li*p47 (145), *Li*FAF2 (164), *Li*UFD1 (46) and *Li*PUB1 (98) immunoprecipitates as show in Figure 3.

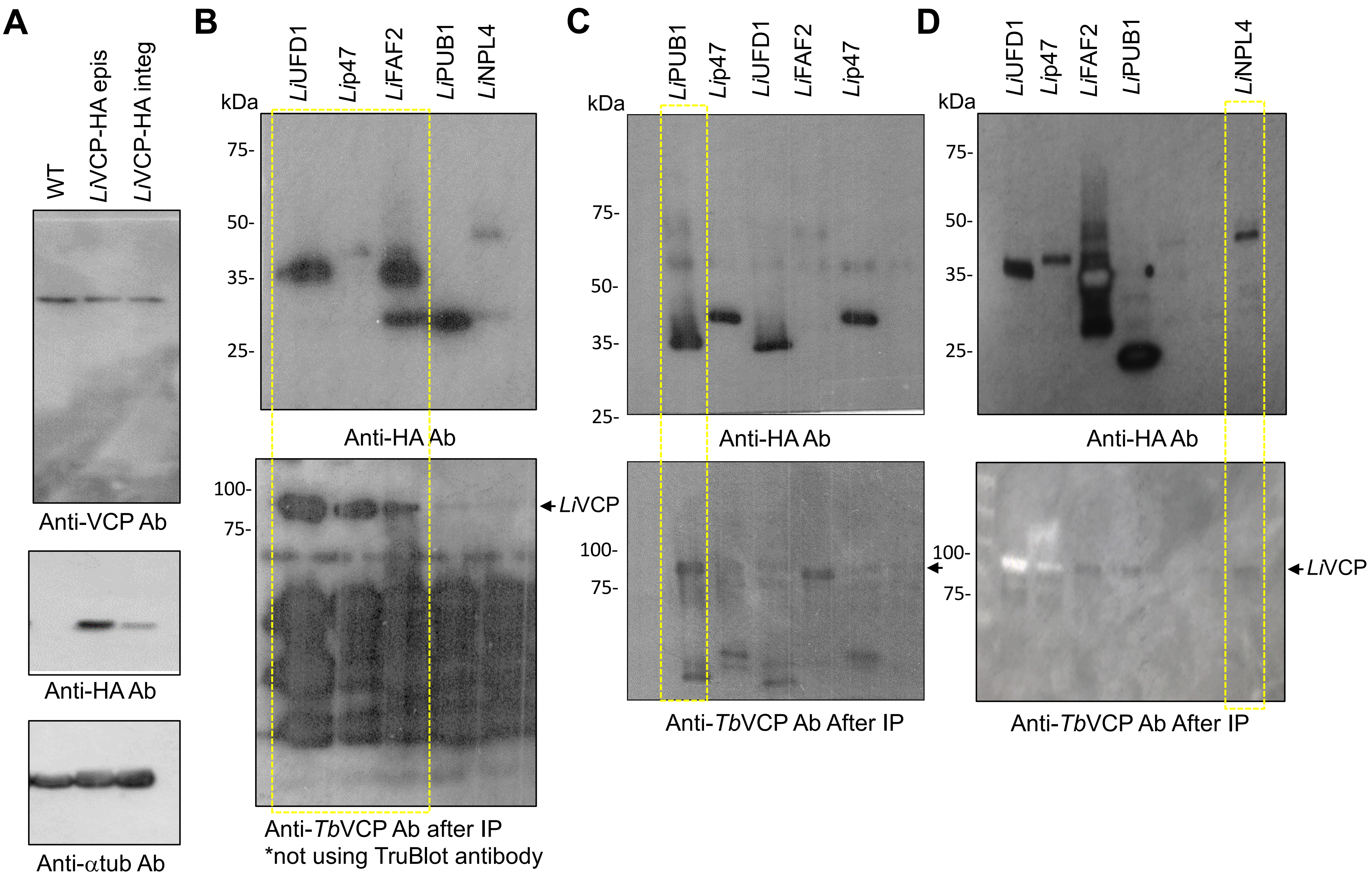

A
WT
*Li*VCP-HA epis
*Li*VCP-HA integ
Anti-VCP Ab
Anti-HA Ab
Anti-αtub Ab
B
*Li*UFD1
*Li*p47
*Li*FAF2
*Li*PUB1
*Li*NPL4
kDa
75-
50-
35-
25-
Anti-HA Ab
100-
75-
*Li*VCP
Anti-*Tb*VCP Ab after IP
*not using TruBlot antibody
C
*Li*PUB1
*Li*p47
*Li*UFD1
*Li*FAF2
*Li*p47
kDa
75-
50-
35-
25-
Anti-HA Ab
100-
75-
Anti-*Tb*VCP Ab After IP
D
*Li*UFD1
*Li*p47
*Li*FAF2
*Li*PUB1
*Li*NPL4
kDa
75-
50-
35-
25-
Anti-HA Ab
100-
75-
*Li*VCP
Anti-*Tb*VCP Ab After IP

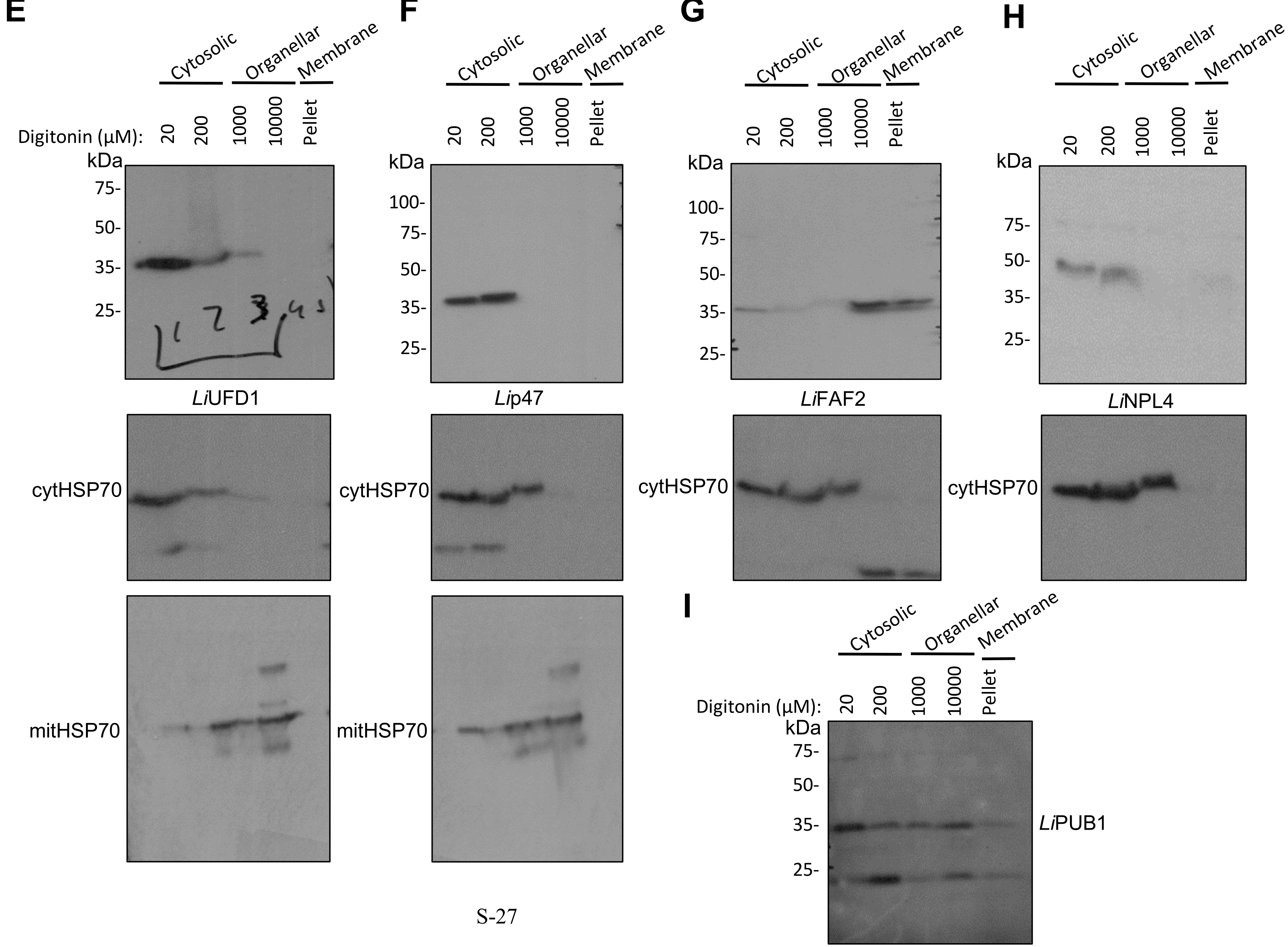

Supplementary Figure S20

Supplementary Figure S20. Uncropped blots for data shown in Fig. 1B (panel A), Fig. 2A (panels B-D) and Fig. 4A (panels I-E). Figure 2A contains blots (cropped) that are derived from different gels. Here in panels B-D, we show the uncropped blots for these data. Highlighted with yellow dashed lines are the corresponding data (but uncropped) shown in Fig. 2A. Panels E-I indicate the uncropped blots for the digitonin fractionation data shown in Fig. 4A.
